# Beyond Blueprints: Exploring the influence of social science factors on engineers’ flood infrastructure design decisions

**DOI:** 10.1371/journal.pone.0345154

**Published:** 2026-03-27

**Authors:** Elham Ajorlou, Mohammad Pourmatin, Ali Farhadzadeh, Majid Ghayoomi, Elizabeth Hewitt

**Affiliations:** 1 Department of Civil Engineering, University of New Hampshire, Durham, New Hampshire, United States of America; 2 Department of Technology and Society, Stony Brook University, Stony Brook, New York, United States of America; 3 Department of Civil Engineering, Stony Brook University, Stony Brook, New York, United States of America; Tecnologico de Monterrey, MEXICO

## Abstract

Communities rely on infrastructure for daily activities, and when assets are damaged during a disaster, they face significant costs, both in physical damages as well as quality of life disruptions. Most engineering research focuses on material properties and modeling to understand the outcomes of nature-infrastructure interactions; however, little research explores the human factors driving engineers’ design decisions. The objective of this exploratory qualitative work is to examine the role of human decision-making among engineers to better understand influential factors for flood infrastructure design. Open-ended interviews were conducted in 2022 with flood infrastructure engineers in the US who work on levees, dams, and breakwaters to better understand design parameter interactions with personal attitudes about risk and climate. Findings indicate four crucial areas that influence the design process: 1) Stakeholder involvement and influence; 2) Risk perception and tolerance of the engineer (and, specifically, tensions with the norms for risk in the home organization and the profession at large); 3) Personal characteristics of the engineer (such as their attitudes toward disasters based on personal experience), and 4) Key areas of weakness within the profession as a whole. This work provides a foundation to advance initial qualitative findings, and takeaways from this research can forge stronger interdisciplinary collaborations, while the regional focus on the U.S. may necessitate caution in global generalization.

## 1. Introduction

The number of annual disasters in the U.S. causing damages that meet or exceed $1 billion is steadily increasing [1]. Collectively, the billion-dollar disasters that occurred over only a 5-year period from 2019-2023 led to nearly 2,000 fatalities and represent almost 23% of the total disaster-related costs incurred in the United States since 1980 [1]. Importantly, local infrastructure assets that are disabled or destroyed during these events represent a significant portion of this cost, both in physical damages as well as disruptions to the community. Many of the vulnerabilities faced by the built environment involve human factors – in terms of the initial design, maintenance, or repair of community assets, but given the inherent subjectivity of these variables, they are often not well quantified in disaster planning.

Engineers play a significant role in resilience of critical infrastructure, as their decision-making and design processes directly influence the vulnerability and adaptive capacity of these systems. Their ability to integrate resilience principles into design—considering factors like risk management, adaptability, and sustainability—ensures that infrastructure can withstand and recover from unexpected shocks. The U.S. Army Corps of Engineers’ guide to resilience practice [2] emphasizes that engineering resilience involves planning, standards, and construction techniques that enhance the ability of infrastructure to absorb damage without complete failure. Engineers’ perceptions of resilience are often shaped by organizational norms, institutional constraints, and risk assessment methodologies, which can influence the robustness of infrastructure systems [3,4]. Consequently, a comprehensive and community-centered approach to resilience is needed to strengthen preparedness and response efforts and guide recovery efforts, while also expanding engineers’ understanding of resilience to incorporate broader social, environmental, and long-term adaptive considerations.

In this study, infrastructure refers to the physical and engineered systems designed for flood protection and risk mitigation, including levees, dams, and breakwaters networks. However, we acknowledge that the engineering community itself is a form of infrastructure – the tacit, social, and cultural processes that shape the practice. While we do not explore this conceptual framing in depth in this paper, it presents a path for future work. Although infrastructure itself is static, infrastructure systems are operated and influenced by social actors and their decisions [5,6,7], which in turn are driven by the interaction of psychological characteristics (of both engineers and end users) and larger socio-technical dynamics. Social science has made significant inroads in technical areas of research, with many science- or engineering-driven fields—energy, for example—now incorporating insight on occupant behavior, social norms, habits, values, and attitudes [8,9,10,11,12,13,14,15,16]. For instance, Allcott [8] found that comparing a household’s energy consumption to neighbors was successful in reducing energy use by 3–5% per household; thus, the field’s understanding of energy from a technical perspective was enhanced through inclusion of a social aspect. Many fields are advancing research in similar ways through collaborative exchanges across disciplines and funding agencies are emphasizing convergence research that merges typically disparate fields [17]. However, despite the recent shift towards interdisciplinarity [18,19] and the dominance of resilience discourse across disciplines, engineering research is still heavily focused on the physical aspects of infrastructure. Thus, a more human-centered research path, merging engineering with social science, may offer deeper insights [20].

To that end, this work undertook a comprehensive set of qualitative interviews with the engineering community (specifically those who deal with flood protection systems) as part of a larger project to better understand how a social science approach to understanding engineering practice can shed new light on design outcomes for resilient infrastructure. More specifically, this work is framed around the following primary research question: ***What are the emergent sociological and psychological themes driving the design process for flood infrastructure and how do engineers conceptualize, incorporate, and process these themes in their design decisions?***

It is important to note that this study is not a conceptual exploration of qualitative approaches in infrastructure engineering. Instead, it uses qualitative methods to empirically examine engineers’ perceptions of water protection infrastructure design, with a focus on how social factors—such as norms, behaviors, and decision-making—shape design outcomes.

The remainder of this paper is organized as follows. First, a framework for human behavior is introduced, including background on psychological constructs that may be relevant to engineering practice, and gaps in current research are outlined. Next, the methodology and theoretical framework for conducting this research is reviewed. Finally, results are introduced and explained, followed by a discussion of main findings.

## 2. Background and related research

Engineering research and design require creativity, problem-solving, and critical thinking [21]. Human behavior, which is driven by a range of individual characteristics, including values, norms, attitudes, and intentions, plays a substantial role in engineering successes or failures [22,23]. Recently, there has been an increased focus on integrating qualitative characteristics of individuals into engineering research [24] through various methods, including user studies, focus groups [25,26], interviews [27] and surveys [28], with a growing recognition of the significance of social science in traditional engineering. Some key areas will be reviewed below.

It is important to note that the focus in this work on the micro-scale individual engineer is interconnected with macro-scale societal dynamics. This feedback loop highlights the influence of societal norms on individual attitudes and the impact of individual attitudes on society, indicating areas for further research [29].

### 2.1. Psychological characteristics of engineers

Communication and collaboration play a significant role in successful engineering projects [30,31]. Studies have shown that effective communication and collaboration among project team members may lead to better decision-making, improved project outcomes, and increased job satisfaction [32].

Furthermore, engineering decisions are influenced by personality traits [33] and normative design analysis [23]. According to the literature, certain personality traits, such as conscientiousness and agreeableness, are associated with successful engineering outcomes. For example, conscientious engineers are typically more organized and trustworthy, which are essential traits for effective communication and teamwork in engineering projects [34,35].

There is also a relationship between job satisfaction, engineering decisions and patterns of social interaction that emerge among engineering team members [36]. Research has shown that engineers who are satisfied with their jobs are more likely to be committed and motivated, work with a positive attitude, and make better decisions [37].

Engineers, as humans, act with bounded rationality, and are subject to various cognitive limitations and biases that can result in poor decision-making [38]. Bounded rationality is a conceptual framing that arose as a divergence from traditional neoclassical economics in order to make sense of the limitations to human rationality [39]. For instance, confirmation bias describes people’s propensity to favor information that supports or validates their ideas or values and is hard to remove once it has been formed [40]. This bias can lead engineers to overlook crucial details that contradict their beliefs, while the sunk-cost fallacy can lead them to persist with a failing project. Importantly, bounded rationality acknowledges that decision-making is influenced by cognitive limitations and biases. While engineers, like all individuals, are primarily educated to make rational design decisions [41], they are subject to inherent cognitive constraints that can impact the decision-making process. In their interview analysis, Dringenberg et al. [41] compared the shared beliefs of two groups—students and faculty—regarding engineering decision-making. Both groups identified rationalism as a core engineering norm and acknowledged that empathetic approaches are generally not prioritized in engineering design decisions.

Motivation and creativity are also crucial personal factors in the success of engineering research and design projects. A study by Brewer [42] found that highly motivated and creative individuals were more likely to report higher levels of inventiveness and problem-solving skills in their engineering research projects. Another growing area of engineering research is the interaction between humans and technology [43] including exploring how people perceive and process information and make decisions. Indeed, human factors engineering [44] in systems design can dictate how people interact with technology and design systems that are easy and safe to use [45]. Importantly, a negative attitude towards a particular type of technology or approach may limit the engineer’s ability to consider it a viable option. Personal biases or prejudices can also influence the design or research, potentially leading to discrimination or other adverse outcomes.

### 2.2. Values, norms, and attitudes

Other psychological characteristics of the engineer that can shape behavior include values, social norms, and attitudes. Significant research exists on these attributes across numerous fields, but little work has connected them to engineering [46,47]. Additionally, each of these characteristics is conceptually distinct, and it is worthwhile to distinguish them from one another here. Importantly, the link between values, norms, and attitudes and related behaviors or actions is particularly relevant.

Values are relatively stable elements of an individual’s worldview [48]. People’s values reflect their beliefs on what they find to be significant and worthwhile in life, and are mental representations of fundamental motivational aims, stable across time and contexts [49]. However, more recent work by van der Weij et al. [50] highlighted that values in design process are not statics but they are dynamic and change over time, for example sustainability has been a dynamic value in the design of energy technologies but emerged over time by the growth of other technologies.

From the perspectives of psychology, sociology, and decision science, values have historically been seen as fundamental components of the self-concept and, accordingly, as a type of “basic truth” about reality [51]. A person who emphasizes values around success, for instance, will likely let those values drive their career decisions (e.g., selecting what is perceived as a highly respected field), their career preparations (e.g., devoting significant time and energy to education and training), and their actions while on the job (e.g., taking on extra work and applying for promotions when possible). Researchers have found a strong connection between values and subsequent actions when a value is suddenly highlighted due to an acute or important event. Therefore, action-value links can be formed through the influence of some situational changes, such as real events (hurricanes) and technological and environmental changes that have the potential to activate some new or temporary values. The characteristics of values affect people’s decisions and serve as a powerful driver for action [52]. Therefore, from the perspective of engineering behaviors, it could be argued that engineers’ and other actors’ values orientation may influence on-the-job decisions that ultimately impact infrastructure outcomes; more work is needed in this area.

The impact that social norms have on behavior has been the subject of extensive research. Social norms are generally defined as an individual’s perception or belief about acceptable behaviors of other individuals within their peer group [53,54,55,56,57]. Social norms are impactful mechanisms because humans naturally compare themselves to others and tend to modify behavior to align with the perceived norm [58]. Although social norms may help to explain some behaviors, other non-normative factors can also play a role, such as environmental variables, policy or technology changes, limited choices, and other external factors [59,60].

Attitudes are closely interwoven with social norms [60]. The term “attitude” is used to describe one’s overarching impression of people (including oneself), locations, things, and problems [61]. According to Eagly & Chaiken [62], the most common definition of attitudes in existing research is as an internal psychological tendency that manifests itself as a degree of either support or opposition in the assessment of some object. Expanding these concepts to engineering research, engineers’ pro-environmental attitudes can play a role in their design choices for coastal infrastructure [24]. Those who have stronger pro-environmental attitudes, for instance, may design more sustainable and eco-friendly structures [63,64,27]. Engineers may also have varying attitudes towards innovation, green infrastructure (GI; [65]), and safety [66], which can influence decision-making in these areas. Unlike values, which are relatively stable elements of an individual’s core worldview, attitudes can change and evolve over time.

Collectively, values, norms, and attitudes form the backbone of a number of well-validated causal models of human behavior [67,68,69,70]. For instance, the Theory of Planned Behavior (TPB), one of the most widely tested models, posits that underlying values influence attitudes, and both attitudes and social norms influence the formation of an intention to act, which directly precedes a behavior. While individuals do not always perform the behavior the intention relates to, TPB helps illustrate the causal chain from one construct to the next; this framing is highly relevant for this work, which attempts to better understand how engineers think about, make sense of, and ultimately act on values, attitudes and norms in the context of design practice.

### 2.3. Risk perception

Risk perception is a well-studied concept in psychology and decision science. Generally speaking, there is a large body of evidence that points to the frequency with which humans incorrectly estimate risk [71,72,73,74]. It refers to how individuals assess and respond to uncertain events, often shaped by cognitive biases and social influences. Many risks are socially constructed, based on cultural and social norms [75]. Individuals rely on mental heuristics and biases to comprehend the probability of risk, but this assessment is typically based on an individual’s own worldview, which can lead to inaccurate probability assessments [72]. However, in engineering, risk perception is further shaped by professional norms, regulatory constraints, and industry standards [76]. An engineer’s risk perception can greatly influence engineering design broadly [77] and coastal infrastructure design specifically [78,32]. Those who are more risk-averse may design over-engineered or overly conservative structures, while those who are more risk-tolerant may design less robust and more cost-effective systems [79]. Studies have shown that salience with a disaster – for instance, a firsthand experience with a flood – can considerably impact the risk perception of engineers [80,81,82]. This aligns well with seminal work on risk perception [71,73,74].

### 2.4. Organizational culture and industry norms

Although individual characteristics of engineers play a key role in infrastructure outcomes, individual engineers form the building blocks of engineering organizations such as government agencies, consulting firms, and private construction management entities. Thus, it is worthwhile to explore the multidirectional influence between the engineer and their home organization. Importantly, while organizational culture can be diverse from firm to firm, and individuals can be highly heterogenous within firms, there are norms within industries that remain relatively stable across different organizations with the broader field (e.g., engineering) [83].

Organizational norms [84] considerably impact engineering research and how engineering projects are managed and executed [85,86,43]. For example, research has shown that organizations with a robust safety culture are more likely to deliver high-quality, low-risk engineering projects [66]. Cialdini & Jacobson [87] highlight that the presence of social and organizational norms indicates the importance of interdisciplinary behavioral analysis research, and engineers are not an exception since social norms impact climate related design decisions. Positive descriptive norms—how others behave in a given context—that drives behaviors can motivate more sustainable design decisions for civil infrastructures if the designers are exposed to them [88].

Prentice & Paluck [89] emphasize the importance of social norms in group dynamics when considering engineering behavior, rather than focusing solely on individual norms. In this context, diversity also plays a significant role in the engineering process. Research has shown that diversity within engineering teams can lead to more creative solutions [90–92]. This underscores the critical role of leadership in engineering groups, as effective leadership ensures that projects are completed on time, within budget, and meet the expected quality standards [93].

Additionally, plans and policies made within organizations play an important role in raising the presence of ethical and moral attitudes and behaviors of engineers to consider the financial and ethical consequences of the design decisions in their decision-making process according to Barros-Castro et al. [94].

### 2.5. Grounded theory background

To explore theories related to the above-mentioned aspects of human actions, grounded theory serves as a valuable approach for analyzing qualitative data. Grounded theory has evolved significantly since its inception, with recent literature exploring its application across diverse fields that emphasizes its methodological robustness and examines its integration with other research approaches [95]. Researchers have applied grounded theory to various areas. In behavioral theories and educational research, studies underscore how grounded theory facilitates the exploration of dynamic interactions and evolving behaviors within educational settings [96,97]. Grounded theory has also been extensively employed in health-related research to investigate health behaviors and patient experiences [98]. In organizational research, grounded theory has played a critical role in understanding complex organizational dynamics. Martin & Turner [99] laid the foundation for using grounded theory in organizational contexts, which has been further expanded by [100] and Assoratgoon & Kantabutra [101] highlighted grounded theory’s capacity to uncover underlying norms and cultures within organizations.

More recently, grounded theory has gained attention in engineering research. Khiat [102] highlighted its emerging role to address complex, technical problem-solving processes. Osman et [103] applied grounded theory to civil engineering design practices to explain essential processes that engineers use to justify design decisions. Hoda [104] introduced an adapted methodology for software engineering research that expands grounded theory’s philosophical foundations and provides flexible methodological steps to accommodate socio-technical contexts. Grounded theory’s adaptability extends beyond traditional social sciences, as seen in its application to green construction, project management, and engineering education [105,106] Additionally, emerging technologies like large language models have been integrated into grounded theory research, enhancing qualitative data analysis and theory development [107].

Recent applications of grounded theory in infrastructure design have yielded valuable insights into flood defense systems, including dams, levees, and breakwaters. Researchers have employed this methodology to examine the socio-economic impacts of levee and dam failures in the Mississippi River region [108]. Grounded theory has also been instrumental in exploring the role of localized risk knowledge and the emergence of new social networks in flood risk communication within vulnerable communities [109]. Similarly, Hirschfeld and Hill [110] utilized grounded theory to assess the alignment between climate service guidance materials and the practical needs of local engineers addressing sea-level rise. These studies illustrate the broad applicability and adaptability of grounded theory across disciplines, emphasizing its capacity to generate robust, data-driven theories in both traditional and emerging research contexts.

### 2.6. Evolving engineering design approaches

Given the increasing complexity of urban systems, contemporary flood defense infrastructure design has shifted toward holistic, cross-disciplinary approaches that integrate urban planning, landscape architecture, and engineering. Addressing urban resilience requires frameworks capable of managing uncertainty and complexity; in this context, scenario-based approaches have been proposed to support strategic responses to multivariate and dynamic environmental challenges [111]. Such frameworks align with the growing emphasis on balancing ecological, cultural, and safety goals while accommodating diverse stakeholder values [112].

The integration of cross-disciplinary expertise further enhances the design process. Collaborative efforts among engineers, landscape architects, and environmental scientists facilitate the development of nature-based solutions (NBS) supported by effective communication and stakeholder partnerships [113]. This collaborative dynamic extends to the role of urban planners, who act as intermediaries between technical experts and community stakeholders, helping to bridge gaps in environmental decision-making and promote ecosystem-based solutions [114]. Yet, professional relationships are shaped by underlying academic cultures; for instance, research engagement barriers persist within landscape architecture [115]. Engineering education further influences sustainable design decisions which emphasizes the need to equip engineers with sustainability informed knowledge [116]. Specifically, in equitable infrastructure asset management for flood-prone areas, incorporating social vulnerability and equity enhances decision-making efficiency and promotes more sustainable design solutions, particularly with advancements in new technologies [117].

In parallel, integrated landscape approaches have been shown to address both societal and environmental challenges effectively, highlighting the importance of cross-sectoral collaboration in fostering resilience [118]. GI has emerged as a core strategy within this integrated framework which enhances urban flood resilience through adaptive planning and ecological design principles [119,120]. Multifunctional urban landscapes not only defend against floods but also support biodiversity and community well-being and highlights the importance of transcending traditional engineering paradigms [121]. Traditionally, cities relied heavily on gray infrastructure designed for efficiency and durability, yet often neglecting ecological integration and climate adaptability [122]. In contrast, GI leverages natural systems, including wetlands, green roofs, and urban forests, to manage stormwater, reduce runoff, and mitigate flood risks [123]. Recent studies have shown that integrating green and blue infrastructure with traditional gray infrastructure enhances flood defense effectiveness while reducing lifecycle costs [124,125]. Coastal protection strategies have evolved from constructing massive seawalls to using NBS like restored dunes and mangroves, that absorb wave energy and reduce erosion [126, 127].

In the US, policy initiatives and engineering advancements have accelerated GI adoption, particularly for stormwater management, flood mitigation, and urban heat reduction. Philadelphia’s Green City, Clean Waters initiative exemplifies this shift, replacing conventional stormwater systems with green solutions to meet regulatory requirements and improve urban livability [128]. Coastal cities, facing sea-level rise threats, have also adopted hybrid approaches. For instance, Hoboken, New Jersey, implemented GI through design competitions to enhance flood resilience [120].

While technological advancement and policies play roles in this shift, engineers’ beliefs, perceptions, and decision-making behaviors significantly influence the adoption of GI in flood protection design. Risk perceptions of and uncertainty often shape their reluctance to adopt GI. Olorunkiya et al. [129] found that professionals perceive GI as riskier due to variability in performance and maintenance needs compared to conventional systems. This perceived uncertainty can reinforce a status quo bias, where familiar gray infrastructure solutions are preferred despite long-term sustainability benefits offered by GI [130]. Moreover, subjective norms and professional culture significantly influence engineers’ decisions. Drescher & Sinasac [131] highlight how younger engineers are more receptive to GI due to recent educational exposure to sustainability, whereas senior professionals often rely on established gray infrastructure paradigms. Institutional inertia and the absence of GI-focused engineering standards further reinforce these biases [132]. Furthermore, social influences, including peer pressure and leadership endorsement, can facilitate or hinder GI implementation. When senior decision-makers advocate for GI, adoption rates increase, particularly in municipalities with participatory planning cultures that connects scientists with stakeholders and policy-makers [133,134]. Similarly, stakeholders’ personal experiences with past flood events shape their receptiveness to novel solutions [135].

These studies underscore the evolving role of engineers, planners, and designers in flood defense infrastructure that advocates for integrated design behaviors informed by ecological and environmental principles for adoption to evolving engineering design approaches.

### 2.7. Gaps in research

Despite these advancements in incorporating human behavior into engineering research, gaps still exist. Previous studies have highlighted the importance of communication, collaboration, cognitive limitations and biases, and organizational culture and leadership in engineering decision-making [136,137,138]. However, there is a lack of research that examines how these factors link to key psychological constructs such as values, norms, attitudes, and risk perception [139,140,141,142]. Notably, the role of training in engineers’ design decisions as a reflection of cultural and educational norms has received insufficient attention [116]. Equally important, how these constructs manifest specifically in coastal infrastructure decision-making remains underexplored [134,141,143], which is a key contribution of this study.

Furthermore, there is a need for in-depth case studies analyzing how internal organizational norms and leadership influence engineers’ openness to adopting green infrastructure solutions [144]. While social learning and peer influence are recognized as important drivers of professional behavior, their specific role in shifting engineering practices toward GI remains insufficiently studied [145,134]

Additionally, while community engagement has been widely studied in flood risk management [31,146], there is a critical gap in research on how engineers themselves engage with communities in the flood infrastructure design process [147–149]. Current literature primarily focuses on community perceptions and responses [150,151,152], yet less attention has been given to how engineers integrate stakeholder input into their decision-making and risk assessment processes [153,154]. This highlights the need for further exploration into how practitioner-driven risk assessments align with or diverge from community priorities and how engineers navigate community involvement in design decisions [155,156]. Despite engineers’ significant role in coastal infrastructure risk assessments, few studies engage engineers directly in the research process, further emphasizing this research gap.

To address the above gaps, this work builds upon recent advancements in engineering research [157] to contribute to the field’s qualitative understanding of engineering practice. This work focuses exclusively on flood infrastructure design and specifically targeted the engineering community (not engineers in research or academic roles), which is a novel contribution to existing work. This work also provides a launching pad for future fieldwork and quantitative modeling that can ultimately validate the qualitative findings presented here.

## 3. Methodology

### 3.1. Overall approach

Qualitative interviews were conducted to advance this research for the following reasons: First, currently available literature on the variables affecting flood infrastructure risk assessment is primarily of a quantitative nature, with lack of qualitative exploratory investigation [158,159,160]. Second, because risk assessment involves multiple, often interconnected variables, it is challenging to develop a universal quantitative model that accurately captures all these influences [161]. Qualitative interviews can add flexibility in exploring these complexities. Third, because the relationship between the social and behavioral components of flood infrastructures and the factors influencing risk assessment is complex, it is difficult for quantitative research to clearly explain the “how” and “why” of this relationship. Thus, there would be a need for a qualitative approach supplemented by quantitative risk assessment [162,163]. Grounded theory was chosen as an appropriate qualitative method for this study since it is primarily interpretive, seeks to identify ideas and links, and offers rationales for occurrences that are already observed [164]. It facilitates the development of new theories grounded in collected and analyzed data, especially when there is no existing theory about a phenomenon or where existing theories are inadequate due to limited empirical evidence [163]. Theories emerge from the data itself, rather than being shaped by pre-existing hypotheses, ensuring they are deeply rooted in the data, necessitating a reciprocal connection between data and theory [165,166]. In qualitative interview analysis, grounded theory helps researchers to build theories iteratively from participants’ perspectives. In this theory, iterative and constant comparative analysis is the key [167] to compare different interviewees responses and explore common themes or differences. Over time, this process helps to refine themes and categories. Coding procedures in grounded theory follow a stepwise approach that iterates over time: Open coding, axial coding, and selective coding, which will be elaborated in subsection 3.3. Throughout this process, the researcher remains flexible, refining codes and categories as new data emerges, and constantly ensuring that the analysis stays grounded in the data. Researchers also write memos—reflections, notes, or thoughts—to track evolving theories and identify patterns. This versatility motivated the present study to employ grounded theory.

The interview protocol consisted of 14 open-ended questions designed to cover a broad range of topics including engineers’ background and training, the influence of community engagement and owner demands, perceptions of risk, design parameters, and conceptual frameworks for resilience. Since the interviews were open-ended, some participants delved more deeply into certain topics over others. All interviews were conducted between June and October 2022 and were held electronically over Zoom to allow for contact with individuals not within geographic proximity of the authors’ institutions. Each interview lasted approximately 45 minutes to an hour, depending on the direction of the discussion. Interviews were recorded with the participants’ permission. S1 Appendix in S1 File provides a summary of the interview protocol.

This study involves human participants approved by University of New Hampshire and Stony Brook University institutional review board (IRB). All participants agreed voluntarily to be a part of the study and were provided with an informed consent document in writing. In this study, verbal consent was obtained rather than written consent to minimize the administrative burden on participants, as the study posed no more than minimal risk. Participants’ verbal consent was recorded during Zoom interviews with their permission, and this procedure was approved by the IRB with a waiver of documentation of consent.

### 3.2. Participants

Prior to conducting the interviews, approvals were obtained from the Institutional Review Boards (IRB) of the authors’ institutions (approval # IRB2021-00378) and all procedures for the protection of human subjects were followed throughout the research. Recruitment for the study began on 3 June 2022 and ended on 29 September 2022. All participants agreed voluntarily to be a part of the study and were provided with an informed consent document in writing.

Three criteria were considered when selecting participants. Interviewees needed to be 1) engineers; 2) experts in coastal infrastructure, specifically levees and breakwater; and 3) current or former engineers. Given the niche focus of the work, we identified and connected with initial contacts through LinkedIn searches, web research on engineering firms and practice areas, outreach emails, and contacts through members of our team. Once initial contact was made with a preliminary sample, we relied on snowball sampling to gain additional participants. The final sample of interview participants consisted of individuals from private industry, government and other professional organizations. We conducted outreach to experts nationwide and 18 volunteers were ultimately recruited for this study. Interviews were analyzed iteratively, and thematic saturation was reached when additional interviews no longer produced new categories relevant to the model structure. S2 Appendix in S1 File provides a summary of participant characteristics.

### 3.3. Analytical approach

Qualitative analysis rests heavily on the comprehensive and robust coding of data [168,169]. Codes were derived in two ways: first, from participants’ responses to the open-ended interview big picture questions (listed in S1 Appendix in S1 File), with multiple codes assigned when necessary to capture diverse aspects of the answers; and second, from themes that emerged spontaneously during discussions. Additional codes were refined and added through an iterative process guided by analytical memos for each interview. To develop a robust and validated set of codes, two coders analyzed and coded the qualitative data through an iterative process to introduce novel theoretical possibilities by dividing written content into specific portions and creating labels [164].

**Fig 1:**
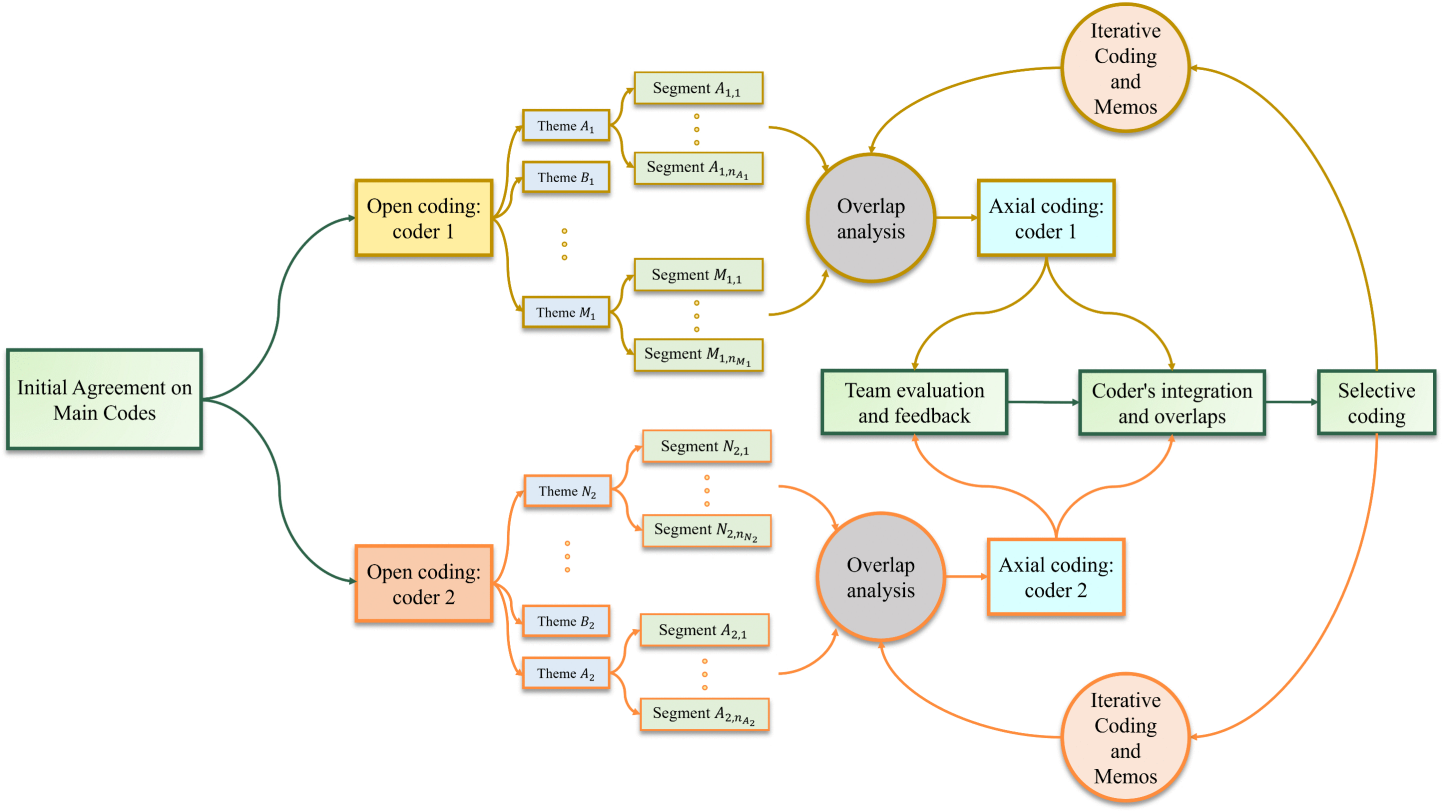
Schematic coding process in this study.

Table 1 presents an example codebook of the most frequently assigned codes derived from the first step of analysis, along with related information, example quotes, and corresponding big picture questions. These labels/codes capture perceptions, actions, impressions, differences, opinions, and other concepts relevant to the targeted research question. The key connections identified among categories formed the primary results of the study and constituted the core analytical process. Finally, all categories developed in the previous steps were integrated and organized around a central core category. The coding process is schematically represented in Fig 1. Detailed procedures are presented in S3 Appendix in S1 File.

**Table 1:** Most frequent jointly integrated codes from open coding by both coders, with example quotes and corresponding interview questions (Q#).

Code	Description	Examples of original statements	Q #
Motivations	Engineers’ personal rationale for working on flood infrastructure	*“This field is highly interdisciplinary, and around every corner there is something that you do not know. Public safety is an integral aspect of civil engineering, which further adds to its appeal.”*	2
Hindrance	The engineers’ personal reasons for feeling less motivated in their work.	*“Submitting proposals is often a time-consuming and unproductive activity, yet it is necessary to secure work.”*	2
Collaboration	Views on working with peer engineers and other stakeholders in flood infrastructure design	*“Effective communication is a key aspect of the job, as it involves liaising with both owners and clients, some of whom may have varying degrees of familiarity with the subject matter.”* *“We collaborated extensively with biologists and environmental experts, which provided me with valuable insights into these fields.”*	3
Interorganizational communication	Engineers’ views in the exchange of information, ideas, and messages as well as challenges between different organizations or entities.	*“There has always been a war between the federal government and state governments; the federal government wants the states to handle their own flood risk. And states want the federal government to handle it because it is expensive.”* *“Communication is the biggest problem we have. It’s the hardest thing we do. So, I think communication is the weakest part of our education.”*	3
Tracking the project	The importance of closely following the progress of a project the engineer has designed	*“The importance of public safety can vary between contractors as compared to engineers, as contractors may not have the same level of obligation towards ensuring public safety.”* *“We are responsible for both construction and post-construction monitoring, which I find to be highly enjoyable and rewarding.”*	4
Community engagement	Personal views on the necessity and influence of community engagement in the flood infrastructure design process.	*“We are typically not heavily involved in that aspect. It is typically the owner who takes the lead, and we may only be asked to attend public meetings or events. However, often the owner prefers to handle such matters themselves.”* *“Community engagement and outreach are crucial components of some of our projects. In some cases, it is mandated by grant funding that supports the project. In other cases, it is simply an essential element in developing an effective solution for the project.”*	5
Regulatory	Opinions about potential changes or modifications to codes and standards	*“There are no standards for printing aquaculture structures in the ocean.”* *“The operation of dams is subject to a multitude of dam safety regulations.”* *“The regulatory framework is undergoing a transition from a conventional standards-based approach to a more risk-informed approach.”* *“The objective of the most recent ASCE 7 update was to enhance safety levels by increasing the design flood standard to 500 years, which was widely agreed upon. However, during the document’s drafting, the higher committee refused the proposal, citing that the cost was too high. This resistance within the engineering community towards improving safety indicates a significant challenge in implementing new regulations.”*	6
State-of-the-art	Engineering opinions about major divergences between the state-of-the-art potential vs. actual practice regarding flood infrastructure design	*“In Dam Safety Engineering, the integration of risk analysis is becoming a significant trend and gaining momentum.”*	7
Training	Engineers’ perspectives on essential training, training shortages, challenges, and improvements, as well as their impact on decisions within flood control projects	*“I would think probably our weakest link right now is how do we educate our future generation who want to take on these infrastructure topics.”* *“Here is a big gap between what you learn in school and then what you will use day to day in practice.”*	13
Maintenance	The importance, urgency, and challenges in maintenance of flood control infrastructures in engineers’ viewpoint	*“In terms of infrastructure, I would say the weakest link is maintenance.”* *“A lot of times, securing funding for capital construction is easier than funding for monitoring and maintenance.”*	13
Data	The significance of data in assessing flood risks and the required enhancements	*“More data is becoming available from FEMA for the engineering regulations to move to higher level of safety.”* *“Having better data to ascertain performance and reduce the level of uncertainty would enhance resilience of flood control infrastructures.”*	13
Real events	Floods/storms mentioned by engineers as disruptive events influential in future infrastructure design decisions	*“And then Katrina happened in 2005, and the (agency) started really putting a lot of emphasis on flooding.”* *“Katrina changed everything regarding how projects are delivered. Clients expect way more for less and in very less amount of time.”*	2
Uncertainty of design parameters	The engineer’s point of view regarding the most significant uncertainties in the flood infrastructure design process	*“My objective is to forecast the soil strength and water pressures within an embankment.”* *“It (uncertainty) varies from project to project.”* *“Material properties are undoubtedly critical, and although obtaining them is feasible, it requires owners to invest in assessing and eliminating potential risks.”* *“Design flood levels, wave characteristics, velocities, and other comparable hazards are essential factors that need to be considered during the design phase.”*	8
Environmental factors	Participants’ views regarding environmental considerations during the design process	*“One of the significant concerns in dam safety engineering is the impact of climate change.”*	10
Financial issues	Engineering challenges in flood infrastructure design due to funding, budget and expenses as well as lifecycle assessment of projects	*“There is a significant gap in funding for improving the safety of dams and water infrastructure.”* *“We are bound by a stringent timeline and budget, which makes it increasingly challenging to meet our goals.”*	11
Personal risk attitudes	Personal feelings and perceptions on risk levels in infrastructure design	*“Being a good engineer often entails being somewhat pessimistic and anticipating potential issues, such as the weakness of the soil or potential failures.”* *“The (agency) has their own guidance on that, and we follow that guidance*	9, 10
Client/Owner	The influence of various agents/actors in flood infrastructure design	*“It’s ideal when your client also places the same value on public safety, and typically this is the case.”*	5, 11
Weakest link	Personal opinions about the area most in need of improvement in the overall process of infrastructure design	*“Communication is a crucial aspect of dam safety engineering, and it extends beyond just communicating with other engineers to include the public as well.”* *“In terms of infrastructure, I would say it is maintenance.”* *“There is a big gap between what you learn in school and what you will use daily.”* *“Most engineering schools tend to have limited emphasis on multivariate probability, which is the foundation of risk analysis.”*	13

### 3.4. Coding reliability

To ensure coding reliability in this study, two independent coders were engaged, with discrepancies thoroughly examined. Differences emerged in various aspects, including overlaps of codes, formation of groups and subgroups, and number of codes assigned. These discrepancies were addressed through detailed investigations and collaborative reassessments. Despite differences, discussions ensured coding consistency. The Cohen’s kappa coefficient (see [170,171] for more detail) and the coding matrix and detailed calculation and procedure are presented in supplementary material, S4 Appendix and Table S4.1 in S1 File. The Cohen’s kappa coefficient is calculated at 0.55, indicating moderate agreement according to the interpretation guidelines proposed by Landis & Koch [172]. It should be noted that this agreement level was calculated considering segments that were coded by both coders. More detailed calculations for all the coded segments are presented in Table S4.1 in S1 File as well. In this study, comparing codes identified by coders from different disciplines (engineering and social science) underscored the significance of diverse expertise among coders. For example, one coder, who is an engineer, identified segments under the theme “Uncertainty” that the other coder, a social scientist, did not code as uncertainty or any other theme. Conversely, the social scientist coder identified segments under the theme “Resilience” that the engineer coder did not code. These differences highlight the unique perspectives brought by each coder’s expertise. It’s important to note that kappa measurements may vary based on code usage and sample size [173]. In this study, although there are themes like environmental considerations that generated substantial agreement between the coders, it was generally found that one coder tended to assign codes to more segments, which had an impact on the overall score. For example, one coder might divide a paragraph into multiple segments, whereas the other coder might choose only a portion of the same paragraph as a segment. This difference in segmentation can affect the quantified level of agreement, even if both coders agree on the assigned theme. Additionally, segments coded by only one of the coders were included in the calculations. The average percentage of agreed segments is also computed at 0.58 (see formula in S3 Appendix in S1 File).

## 4. Results

### 4.1. Open coding

Multiple iterations of comparing and analyzing the data led to the discovery of 18 distinct codes in this study (examples presented in methodology section in Table 1). A summary of the primary codes is presented in Table 2. This table illustrates the primary codes grouped with related topics the interviewees talked about. According to Table 2, risk analysis, community engagement, and uncertainties were the most frequent topics of discussion during the interviews. To gain a thorough understanding of the subgroups, Table 2 also presents the main codes with their corresponding subgroups. Main codes refer to the overarching themes that align with the core interview questions and were frequently mentioned by participants. Subgroups represent branches that emerge when a participant discusses a main code in more detail. It also outlines the rationale for why each subgroup is associated with its respective main group, alongside the frequency of mentions for each subgroup during interviews when talking about the main group. Additionally, an example quote is provided for each subgroup to enhance clarity regarding the results. For instance, the table illustrates that “public safety” was mentioned 7 times when participants discussed their motivations. Low-level codes support axial coding and provide insights for discussing themes frequently mentioned by participants, which highlights their importance and potential common causes.

**Table 2:** Main codes with subgroups, frequencies, and example quotes, providing insights into why each subgroup is associated with its respective main code. For example, public safety was mentioned seven times in discussions of participants’ motivations, as illustrated by the quote: “Ensuring public safety is my foremost priority in engineering decisions.” Note that the number of segments assigned to each main code does not equal the sum of its subgroups, as the listed subgroups represent key highlighted themes rather than all participant references within each main code.

High-level code (# of quotes)	Low-level code (#of quotes)	Reasoning	Example quote
Motivation (46)	Public safety (7)	Humanitarian Concerns	*“It’s public safety, which is, again, part of the draw of civil engineering.”*
	Multidisciplinary topics (5)	Collaborative Opportunities and Professional Growth	*“I think the most rewarding aspect of it is that it’s multi-disciplinary.”*
	Risk mitigation (4)	Ethical Obligations/ Professional Accountability/ Community Resilience	*“Real goal is being able to put risk reduction or protection measures in place that are going to help people and they don’t have to go through that type of thing.”*
	Interested in ocean/coast (4)	Living Area Preferences/ Personal Passion/ Environmental Connection	*“It’s kind of been one of my passions, living and being close to the coast.”*
	Building economy (2)	Economic Development/ Infrastructure Investment	*“Saving life and saving money. So that’s something that motivates us to work with our clients.”*
	Gaining experience (1)	Career Advancement/ Personal Growth	*“Around every corner there’s something that you don’t know or a field of study that you know nothing about. And it’s just absolutely fascinating to be interacting with all different kind of backgrounds.”*
	Infrastructure safety (1)	Public Protection/ Risk Reduction	*“I’ll probably just making, making dam safer.”*
	Teaching people (1)	Knowledge Sharing/Personal interests	*“That’s what drives me. Making the world a better place. Teaching people to do it.”*
	Protect environment (1)	Environmental Stewardship	*“We tried to help build the economy and we try to protect the environment too.”*
	Making money (1)	Economic Development/ Private Sector Engagement	*“The commercial aspects of it. It’s wonderful to make money.”*
Risk Analysis (133)	Risk perception (12)	Subjectivity in Risk Assessment	*“I would say on the individual engineer level we’re tasked with trying to estimate soil properties. And that’s where being conservative or not conservative can come into play. And you want to be accurate and conservative. I’d like to think that a good engineer is a pessimist in terms of thinking about what can go wrong and how weak can this soil be.”*
	Factor of safety (4)	Uncertainty Management/Design Robustness	*“The whole idea of doing a risk analysis is to quantify, like from my perspective, what these safety factors are.”*
Community engagement (116)	Community education (7)	Risk Awareness and Preparedness/Building Trust and Credibility/Building Trust and Credibility	*“Most of the time or a lot of the time, the community folks, they’re smart folks too and they just have a different set of experiences. And so you have to relate it to the people and how they understand things.”*
	Equity (1)	Inclusive Decision-Making/ Responsive Design Solutions	*“That will be interesting as we go forward to see how we can bring equity into this whole planning phase in the United States and tackle that equity challenge.”*
Uncertainties (106)	Standards (3)	Address uncertainties and mitigate risks	*“The (agency) has their own guidance on that. And we follow that guidance, which involves evaluation of at least in the coastal environments, sea-level change, and incorporate formulating all plans to sea level change based on the planning horizon for the project.”*
	Land use (2)	Land use projection and efficiency	*“And of course there are uncertainties on, what is the land-use going to look like.”*
	Hydraulic model (1)	Modeling Assumptions/ Data Limitations/ Scenario Analysis	*“I would say that that’s one of the uncertainties that I’ve noticed over the last several years, you know, just trying to get these hydraulic models and they’re never going to be consistent.”*
	Flood level (7)	Hydrological Variability/ Data Uncertainty/ Climate Change Impacts	*“When I say unknowns I mean, we have data, we have protections, we have statistics, but it’s still the certainties pretty wide ranging, right? Even from a sea level change standpoint, NOAA has 12 different estimates.”*
	Geometry (1)	Construction Tolerances/ Modeling Simplifications	*“There are so many levels of design alternatives … geometries and configurations influence the set of design alternatives.”*
	Material properties (4)	Site-Specific Conditions/ Geotechnical Variability	*“It is a big deal. There are some materials like steel and concrete that have much more predictable properties until they degrade. But soil and water issues that we deal with in dams have a lot of uncertainty.”*
	Attitudinal uncertainties (3)	Uncertainty in decision-making	*“That’s the social piece uncertainty. How are humans going to behave? And how will they follow evacuation orders? How are our warnings going to be disseminated?”*
	Climate change (9)	Inherent Uncertainty in Future Projections	*“The most crucial uncertainties I would say are related to the climate and the ever-changing environment.”*
Resilience (63)	Sustainability (4)	Resilience prior definitions identified by interviewees	*“The piece of infrastructures ability to sustain that hazard”*
	Adaptability (7)		*“Basically, this is what resilience is about: readiness to trying to adapt to risk.”*
	Technical resilience (2)		*“Setting the design flood elevation right. Not only for current events but looking into the future.”*
	Budget resilience (3)		*“The primary one would be …, the second one would be cost efficiency, because given the length of our coastlines, of assets at risk, of cost efficiency in my mind, becomes increasingly important.”*
	Environmental resilience (1)		*“There is an environmental resilience. That is, if all fails and you get a big cost from an environmental point of view, does that come back and how fast? And can we minimize the rebound”*
	Social resilience (2)		*“We are always doing that the resilience term is helping in a way by increasing awareness of the public, of the stakeholders, of the project partners and the owners to think about that.”*
Weakest link (39)	Construction (1)	Identified Critical Needs and Priorities	*“… we’re really good at building things … but then it’s left to the local community and local owner to maintain it”*
	Maintenance (4)		*“In terms of infrastructure, I would say it’s maintenance.”*
	Uncertainty consideration (1)		*“Reducing the level of uncertainty would help”*
	Technology and data (3)		*“I think in terms of technology, there has been a lot of changes. We are moving in a direction, maybe not as fast as it could be, but it seems like a good start to accelerate. So, I think there is gap in terms of applying the updated technology to the industry.”*
	Budget (4)		*“I think a huge gap we have had in funding or financing.”*
	Community engagement (1)		*“There needs to be a lot of public opinions. I feel that oftentimes we are missing inputs from local stakeholders and what they mean, what are inputs from them, or what would benefit them in addition to just putting up an asset that is predicate a flood or earthquake. I think that that is a gap.”*
	Training and education (14)		*“Right at the beginning of that chain of events to talk about is the education part.”*

### 4.2. Axial coding

Based on Tables 1 and 2, the 18 open-coded categories are grouped into four larger ones, revealing a clear pattern of association. Table 3 presents the axial (linked) codes. The initial codes of *community engagement*, *collaboration*, *client/owner*, and *tracking the project* together constitute the larger code of **stakeholder challenges in infrastructure design**. The engineers’ **risk tolerance and perception** are affected by *financial issues* including *budget, funding, lifecycle assessment,* as well as *regulatory*, *personal risk attitudes*, *environmental factors,* and *uncertainty of design parameters*. The **attitudinal view of engineers** is affected by *motivations*, *hindrances*, *state-of-the-art*, and *real events*. *Training*, *maintenance*, *data*, *state-of-the-art*, and *interorganizational communications* are reported as the **weakest links** in this field. To offer deeper insight into axial coding results, the following paragraphs outline the rationale for the inclusion of specific categories or low-level codes within each high-level theme.

**Table 3:** Axial coding.

Main categories	Corresponding codes	Meaning of categories
Stakeholder challenges of designing infrastructure	Community engagement, Collaboration, client/owner, tracking the project	What are the main challenges in the design and decision-making process when thinking about linkages between community and key stakeholders?
Risk tolerance and perception	Financial issues, regulatory, personal risk attitudes, environmental factors, Uncertainty of design parameters.	How does individual risk alignment influence the design process, and how engineering risk tolerance is affected by other factors?
The attitudinal view of engineers	Motivations, hindrances, state-of-the-art, and real events.	How do personal behaviors and norms influence the design process?
Weakest links	Training, maintenance, data, the state-of-the-art, interorganizational communications	From an engineering view, where is the most crucial place to focus on to strengthen the resilience of infrastructure

**Stakeholder challenges of designing infrastructure:** This high-level theme delves into the complexities surrounding the design and decision-making processes, particularly regarding linkages between the community and key stakeholders. This theme emerged from discussions focused on identifying the primary challenges encountered in these processes. Low-level codes such as community engagement, collaboration, client/owner, and project tracking contribute to this theme.

- ***Community engagement:*** Community engagement emerged as a significant challenge, with participants emphasizing the need for community education to facilitate connections between the community and stakeholders. Participants noted the importance of outreach and education initiatives to address challenges resulting from a lack of community understanding of risk. Planners have recently played a greater role in these efforts. However, despite recognizing the importance of planners, they were rarely mentioned as facilitators, reflecting organizational variability—*“the owner usually takes the lead on that”*.

Some engineers expressed a desire for direct engagement, highlighting a tension between current organizational practices and personal preferences for deeper involvement, as the influence of the community is sometimes underestimated.

*“It’s very important for planners and engineers not to underestimate the power of communities these days to block projects. We need to be present... what we see in central planning may not match reality on the ground.”*

Participants’ perceptions also suggest that what planners convey between the community and engineers may not always align with reality. However, in certain cases, public outreach is completely beyond the engineer’s control and is sometimes discouraged due to budget constraints. As one interviewee from a government agency explained:

*“In the (organization), basically all the community outreach is done through the project manager, who is the face of the project, while managers discourage (some of the other) community outreach in the private sector.”*

According to participants, one way to effectively communicate flood risk is to connect people’s personal experiences to historical events. Every failure helps people understand risk in a more tangible way. An interviewee from the private sector says:

*“Historical perspective on what happened in that community (after a hurricane, must be collected and involved in future designs, such as) some of the problems that they’re seeing now, maybe the past decisions or other things that might have constrained them in the past.”*

To provide a comprehensive view, we include a detailed account of these perspectives and all supporting quotes in the Supplementary Materials (S5 Appendix in S1 File).

- ***Collaboration:*** Collaboration in designing flood control infrastructure emerged as another key aspect within this theme. According to interviews, while collaboration is deemed necessary due to the multidisciplinary nature of such projects, it also presents challenges. Participants highlighted issues such as communication barriers and differing levels of understanding among stakeholders. Interviews reveal diverse perspectives among engineers regarding interdisciplinary collaboration and the role of planners. While participants generally acknowledged the importance of engaging multiple stakeholders:

*“So absolutely, we need to get all the perspectives at the table to solve some of these challenges related to technical subject matter experts, we needed that people that work on the policy, we need the planners, the environmental scientists, can’t be solved alone.”*

Engineers from smaller firms described challenges since individuals are assigned to multiple roles instead of interdisciplinary collaboration:

*“You are the coastal engineer, you are the geotechnical engineer, you are the structural engineer,... honestly a little stressful.”*

The role of social scientists in engineering projects was recognized by several engineers, who acknowledged the potential value of collaborating with them. One participant noted, *“I really think that going into the design process, you need to have the social and cultural side.”* While such collaborations are emerging in some organizations, they are not yet fully integrated into standard practices. For example, one participant remarked *“So if you look, it’s like some of the social science in (the office), we realized that the uncertainty on the consequences piece was as large as everything else we’re working on. We looked around in the 35 thousand people in (the office), and we realized we had three social scientists.”* This reflects the growing recognition of the value of social science, though its integration remains inconsistent across contexts.

- ***Client/owner:*** While engineers ideally expect alignment with clients and owners on the prioritization of public safety, interview data reveal variability in how stakeholders influence risk-related decisions, that impact public safety. This variability is often shaped by institutional norms. For example, private sector owners frequently prioritize cost efficiency over risk mitigation, driven by profit motives and budget constraints. In contrast, government agencies may demonstrate a greater willingness to invest in long-term risk reduction, which is a tendency influenced by regulatory oversight*—“The federal guidelines for dams … have not changed in a long time”*.

The pressure to meet stakeholder expectations often forces engineers to balance technical accuracy with project feasibility. One interviewee highlighted the challenge of navigating diverse client expectations, stating:

*“You have to be able to talk with some of those folks. Some of the more sophisticated clients understand it, but you do have to deal with some folks who are all money guys… you are doing engineering in-house because they do not have any money, and we are just trying to help them and that kind of thing.”*

This reflects how financial limitations and varying levels of stakeholder understanding can constrain engineers’ ability to fully integrate risk considerations into design decisions. To navigate these complexities, several engineers reported the necessity of *“putting themselves in the owner’s shoes”* as an adaptive approach to client communication. Furthermore, political and economic pressures further complicate the design process. One interviewee noted that in the private sector, political motivation can shape project outcomes:

*“When you work out there in the private sector, you oftentimes have a politician pushing a project, and they want to push the product to a certain outcome in their mind that will maximize their political benefits of that project… there is some pressure to try to make this project work to satisfy the customer. And then there are economic pressures from the side of the customers”*

However, the adaptations mentioned above regarding satisfying the owner and client raises critical questions about potential ethical dilemmas, particularly when engineers feel pressured to downplay risks to satisfy client priorities.

**Risk tolerance and perception:** This high-level theme consolidates various low-level codes that relate to the question of how individual risk tolerance influences the design process and how this, in turn, is affected by other factors. This theme emerged from discussions centered on understanding participants’ perceptions of risk and their approaches to managing uncertainties in engineering design.

- ***Regulatory:*** Building on the connection between organizational factors—linked to the client/owner theme in engineering challenges—and regulatory aspects, another interviewee elaborated on the limitations of existing federal guidance, such as the coastal engineering manual:

*“On the federal side, we have the coastal engineering manual, … I think the last time it was (partially) updated might be 2011. For example, that guidance is missing a lot of the probabilistic context for probabilistic design, uncertainty and incorporation of uncertainty and all that. And you can see that in the application of the equations that it has, there’s a lot of deterministic methodologies and event-based methods, methodologies, right?”*

Interestingly, this perspective contrasts with another interviewee’s more optimistic observation:

*“Our federal agencies have been for several decades on the path of more of a probabilistic approach to how they address risks”.*

The differing views seem to come from having different professional perspectives. The optimistic perspective likely comes from someone actively involved in regulatory development and national-level programs which provides them with an insider’s understanding of the progress federal agencies are making toward comprehensive risk assessment frameworks. In contrast, the first interviewee, working at the application level, experiences immediate challenges associated with regulatory gaps and expresses concern over the lag between policy updates and real-world implementation.

- ***Personal risk attitudes:*** Despite personal risk perceptions, engineers are often bound by organizational norms and regulatory requirements. Participants noted the challenges of navigating regulatory frameworks and the need to adhere to established standards, even if they may differ based on the organization engineers work for.

All of the interviewees agreed that tacit norms about risk in the engineering profession generally drive them toward conservatism. As one interviewee explained:

*“In the private sector, you could basically look as conservatively as you can without blowing up the cost because the main problem with conservatism is cost. So, I would say overall, in the private sector, engineers try to be conservative.”*

According to the interviews, the most significant factors driving risk-averse decisions in flood risk analysis are, first, feeling responsible for people’s lives and, second, being sued. An interviewee in the private sector explained:

*“There’s nothing I work on that can give me an income that covers my economic liability because a levee fails. No one can carry a billion dollars in professional liability insurance.”*

However, others said that because the hazards are very well quantified, there is little room for subjective opinions, and agency and regulatory norms are more influential than personal risk tolerance. As one interviewee explained:

*“The (Agency) has their own guidelines, and we follow those guidelines, which involve the impact of sea-level rise on coastal habitats and adapt our designs accordingly.”*

- ***Environmental factors:*** Environmental assessments and associated uncertainties also play a crucial role in engineering risk assessment and perception. Participants noted that updating standards to reflect new environmental considerations often face resistance during the drafting process, mainly due to concerns about increased costs. This highlights the challenge engineers face in balancing improved safety standards with financial considerations. As one interviewee explained:

*“In the most recent ASCE 7, it was our goal in the update to move to a higher level of safety, that is the 500 year. Everybody agreed. But when we wrote that the document, the higher Committee rejected it because they think nobody should have to pay for that. So there’s resistance even inside the engineering community that dictates these regulations towards improving safety.”*

Interviews revealed a range of perspectives on nature-based and environmentally conscious infrastructure design which highlights both growing awareness and ongoing challenges in implementation. Some participants recognized the value of integrating ecosystem-friendly approaches into flood infrastructure planning. One engineer noted, *“We do look at the ecosystems in the area… we look at water plants and how they interact with the system.”* Another emphasized the importance of considering carbon footprints in design, stating, *“Everything we put out in the ocean has a carbon footprint… we need to take that into account from the beginning to the end of the design process.”*

However, while some engineers advocated for a more meaningful focus on nature-based solutions, others pointed to practical barriers in regulatory frameworks and quantification challenges. One participant explained, *“We know that nature-based features should work to some extent, but quantifying their cost-benefit impact is a challenge.”* These findings suggest that while the concept of infrastructure is evolving, engineers’ ability to integrate green and blue infrastructure into projects remains influenced by technical, regulatory, and economic constraints.

- ***Uncertainty:*** Participants highlighted the uncertainties related to habitat assessments and climate change, which impact engineering risk perception. Data analysis shows that the greatest source of uncertainty in participants’ design process was related to climate change and its insufficient representation in existing regulations. Interviewees noted:

*“The most crucial uncertainties I would say are related to the climate and the ever-changing environment.”*

*“We have tried to deal with those uncertainties with our factors of safety by having pretty large factors of safety. And now we’re trying to be a little bit more explicit with statistics and what we think are the uncertainties.”*

- ***Financial issues:*** Low-level codes such as financial issues play a significant role in shaping engineers’ risk perceptions and decision-making processes. Participants highlighted how budgetary constraints and financial considerations influence the level of risk or risk perception associated with design decisions. For example, the budget available for a project can dictate the design parameters of flood control infrastructure.

**The attitudinal view of engineers:** In exploring the attitudes and personal perspectives of engineers towards their work, several key aspects emerged from the interview data. Participants frequently linked their motivations, demotivation, experiences of real events, and considerations of the state-of-the-art of the field to their personal attitudes and behaviors. This connection was particularly evident when participants responded to questions concerning their attitudinal views and how these influence the design process. Motivation emerged as a central aspect within this grouping. Participants described various motivations that drive their decisions and actions during the design process. For example, one participant highlighted the impact of experiencing a real destructive event, such as a flood, on their approach to design decisions. Furthermore, participants discussed how their personal views and attitudes influence their opinions on the perceived gap between current practice and the state-of-the-art. Based on the results of axial coding and the observed connections between motivation, demotivation, real events, and state-of-the-art considerations, it was decided to group these low-level codes under the theme of “The Attitudinal View of Engineers.” This thematic grouping reflects the underlying influence of personal attitudes and behaviors on engineers’ perspectives and decision-making processes. For instance, an individual’s attitude towards challenges can shape their approach to work and drive impactful solutions in flood-related matters. One interviewee shared:

*“Being engaged in addressing the everyday challenges in flood control projects, being involved with training to address flood issues, and helping people to cope with another disruptive event is just as interesting and motivational.”*

Another engineer highlighted the importance of patience in the engineering process:

*“I think the timing in large infrastructure projects is a demotivation. These projects can take years and society might have a political shift that basically shuts down projects. The timeline in flood control projects is a little frustrating, it takes forever. It’s important to be patient in this industry.”*

Some engineers see collaboration not just as a professional necessity but as a personal value that motivates and satisfies them. This perspective, expressed in interviews, highlights how individual attitudes toward collaboration go beyond the challenges discussed. This was reflected in interviews. As two interviewees explained:

*“It would be very sad if I went through a project, and I didn’t learn something from an idea that somebody else has or an approach they might take since ways to fix flood infrastructure issues can go on a lot of different directions.”*

*“I’m working collaboratively when all the individuals are open. It really leads to a cross-pollination of ideas. And you can build upon the intelligence of your colleagues and the people that you’re working with. Although, I am good by myself for 85 percent success in a project, in order to get to 95 percent success, I always need input from somebody else.”*

A number of the interviewees commented on the personal impact of specific events like Hurricane Sandy, Hurricane Katrina, and other extreme events in pushing designs towards conservatism. As two interviewees explained:

“Having some experience might have influenced you, including seeing actual storms occur or damages that have occurred over the course of your life that impact how you would take those risks into consideration and the level of uncertainty that you are willing to be comfortable with. Personal attitude might stem from experience, a real event that happened.”

“The Indonesian tsunami, Hurricane Katrina, the frequency of disasters, and the impacts of those events made me realize there was a lot of work to be done in this area. Then I started my career in risk assessment of flood infrastructures and communities”

- ***Ethics:*** Ethics were mentioned a few times during the interviews, though not as a central theme. Interviewees acknowledged both social and environmental ethics, but prioritizing these can be challenging for engineers, as their outcomes sometimes contradict. One participant stated:

*“Ethics change over time. And so the engineering sought to learn and adapt with society’s changing ethics. Society doesn’t make decisions today like it did 50 years ago. The primary ethic was to get the water away from the cities as fast as possible …. But environmentally, it was a disaster, Or in South Florida, in the largest swamp in the US, we’ve straightened rivers because they were hard to navigate with all the curves and it was really bad for the environment.”*

Social ethics, particularly public safety, were often cited as a priority. However, engineers also raised important ethical questions about entitlement and fairness, as well as the political and philosophical implications of resource allocation under economic constraints:

*“It’s public safety, which is, again, part of the draw of civil engineering. The licensing aspect of it makes public safety primary as opposed to the legal profession which really has to serve their clients as being primary. Within the legal structure, of course. Our primary obligation is public safety, which is just nice. I like that.”*

*“You should just pour money at us because we’re special and entitled and privileged. So all of this gets to be quite political and philosophical and about what criteria should be changed, should be viewed in a stressful economic environment that has inflation and has conflicting budget constraints that supply chain issues”*

**Weakest links:** This high-level theme centers on identifying the most critical areas for strengthening the resilience of infrastructure from an engineering perspective. This theme emerged from discussions about priority areas that require attention and improvement in infrastructure resilience. Low-level codes such as training, maintenance, data management, state-of-the-art practices, and interorganizational communications are grouped under this main theme. Maintenance emerged as a significant weakness, with participants highlighting the importance of investing in the upkeep of existing infrastructure. Participants noted that inadequate maintenance practices may undermine the effectiveness of infrastructure resilience efforts. State-of-the-art practices versus practical implementation also emerged as a key weakness in infrastructure resilience efforts. Participants highlighted discrepancies between academic advancements and practical applications in engineering design. These disparities pose challenges for engineers in translating theoretical knowledge into practical solutions. Additionally, interorganizational communications were identified as a significant weakness in infrastructure resilience efforts. Participants highlighted challenges such as manipulation behind the scenes and communication issues between federal and state governments. Differences in regulations and approaches across organizations further exacerbate communication difficulties. One participant highlighted the challenges posed by differing risk assessment approaches between federal and state agencies. It is worth noting that while interorganizational issues were identified as significant weaknesses, they were not the primary response when participants were initially asked about the weakest links in infrastructure resilience efforts.

**Fig 2:**
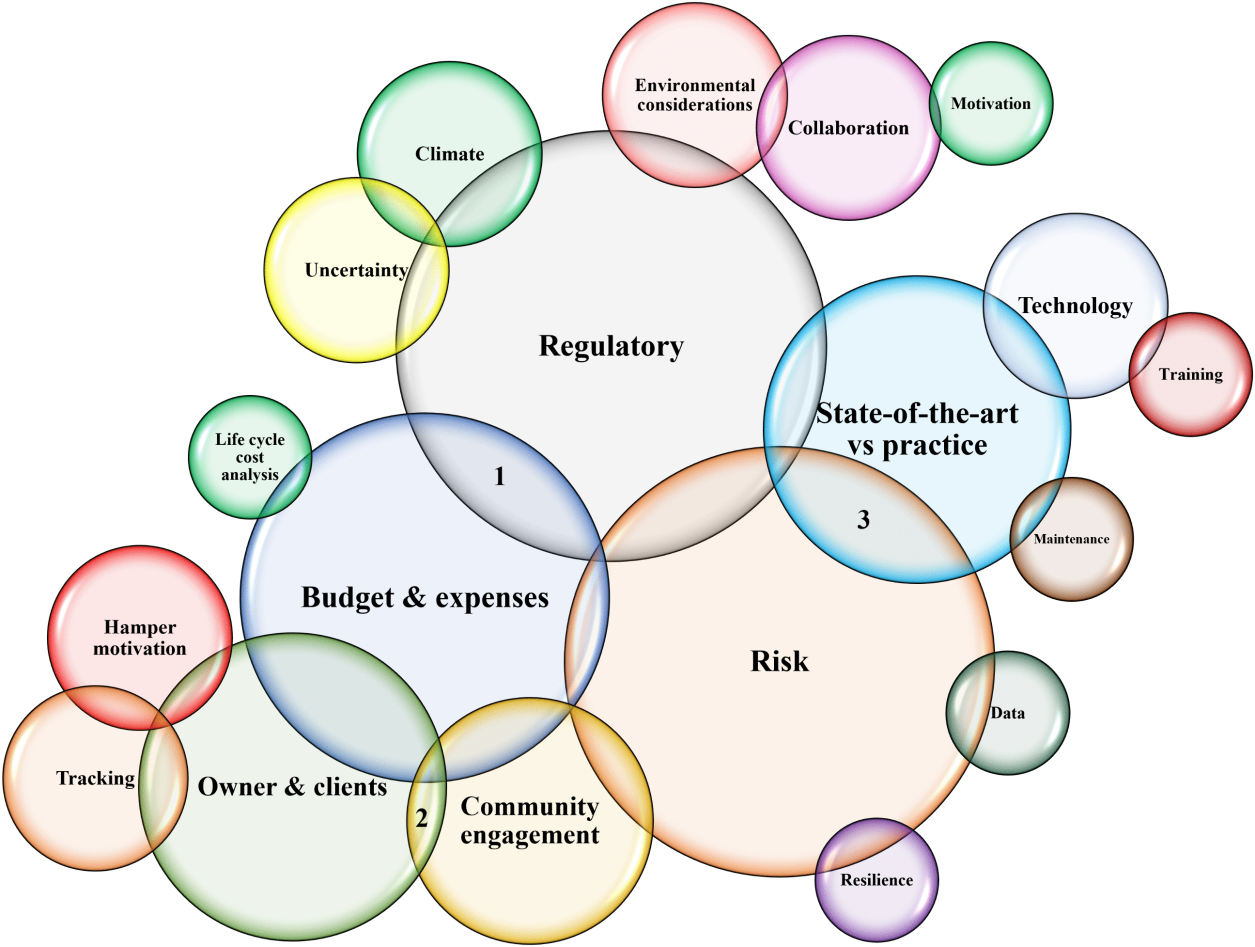
The Ven diagram for the major codes’ overlaps; size of the circles relatively indicates the number of connected codes (e.g., code “Risk” has overlaps with 5 other major codes so that it is relatively a large circle). The numbers on the overlaps refer to the following example quotes: *1. “When the project is authorized, it automatically has a lower budget estimate., then they bring it into designer and say, this was authorized, you need to design it to the proper standard, but we’re not going to give you any more money because it’s only authorized at this lower level. They don’t consult with all the local non-federal partners about specifically some changing criteria.” 2. “There are some public outreach meetings and check ins with the public, which is more or less controlled by the client. The client basically dictates what is the stage they want to get engaged.” 3. “The state of the practice is now moving to risk-based”.*

After labeling interview transcripts, the overlap analysis of the most important keywords such as resilience and uncertainty were plotted in Fig 2. Overlap analysis serves multiple purposes and offers various benefits in qualitative research, as confirmed by existing literature [174–176]. By examining overlaps in interview statements, researchers can identify recurring themes and codes that are consistently mentioned across participants, shedding light on areas of consensus or disagreement [177] as discussed in coding process for qualitative research [176]. The open, axial, and selective coding strategy used in the present study facilitates an ongoing process where the researcher interacts with the data, continuously compares information, and applies techniques to condense and organize it. This dynamic approach allows for the identification, coding, and interpretation of key themes relevant to the research focus. Axial coding establishes connections among open codes to develop core codes. Core codes emerge as combinations of the most closely overlapping open codes with strong supporting evidence  [176,178]. This analysis helps detect patterns and trends in participant responses, facilitating a deeper understanding of commonalities and variations within the dataset [177]. For example, a significant number of participants discussing regulatory aspects in relation to the gap between state-of-the-art and practice suggest a consensus on the need for regulatory updates. Moreover, overlap analysis allows researchers to refine and enhance coding schemes iteratively by identifying emerging themes or codes through the analysis of overlapping statements. This iterative process enriches the coding framework and ensures the capture of all relevant concepts [178–180]. For instance, discussions on community engagement often intertwined with topics related to project owners and clients and the later added to the codes as a new theme, indicating a perceived connection between these entities and the potential for community engagement. Later other initial codes also showed overlap with “owner and clients”.

## 5. Discussion

### 5.1. Main findings

The purpose of this qualitative exploratory study is to investigate and explore factors that influence decision-making among engineers to gain insights into the design and risk assessment process for flood infrastructure.

**Fig 3:**
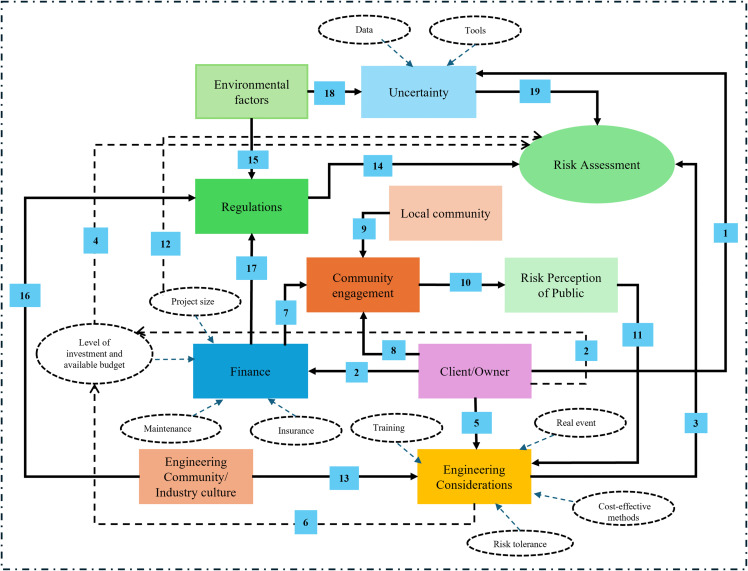
The flow of decision-making and risk assessment for infrastructure design.

The qualitative data analysis highlighted four key themes that significantly affect an engineer’s choices, as highlighted previously in Table 3, including 1) major stakeholder challenges (such as owner and community member influence), 2) risk tolerance and perception, 3) personal characteristics of the engineer, and 4) weak areas in the profession. These factors, in turn, interact with each other and can manipulate risk assessment for public safety. Therefore, decision-making for risk assessment in engineering for flood infrastructure is a dependent variable that various factors (independent variables) can manipulate. Fig 3 depicts a model of this flow of decision-making for flood infrastructure. Each of the four main themes that emerged from the coding process will be discussed in more detail below.

### 5.2. Stakeholder challenges of designing infrastructure

Stakeholder challenges encompass several areas as the original open coding analysis highlighted, including community engagement, collaboration with other disciplines, client/owner involvement, and the ability to track the project from start to finish. As illustrated in Fig 3, our findings indicate that the owner and client can significantly influence how uncertainties are considered and managed in a project (arrow number 1), particularly in terms of acceptable levels of risk for a project and the level of investment deemed acceptable for managing such risks (arrow number 2). For example, in the case of flood protection, the choice of an acceptable flood level for the project may be influenced not only by engineering considerations (arrow number 3) but also by the available budget for the project (arrow number 4).

Some engineers expressed concern that relying on planners for community communication is not fully practiced and may not adequately capture on-the-ground realities. Additionally, individual motivations and personal values also influenced preferences for direct community involvement over intermediary-led engagement. This reflects a tension between organizational practices and individual preferences for deeper involvement, particularly when the significance of community input is perceived as underestimated. These insights suggest that, while planners and managers can effectively mediate, the involvement of engineers in these interactions could lead to different project outcomes and enhance personal accountability in addressing societal challenges.

Interviewees also had valuable thoughts on the role of the local community in the design process of coastal infrastructure, emphasizing that the type of organization (e.g., government, private sector, etc.), project size, and owner dynamics can influence both the extent and the process of community engagement (arrow number 7 and 8), as well as the role of the engineer in engaging directly with community stakeholders. In some cases, public outreach is entirely outside of the hands of the engineer, and at times, discouraged altogether due to budget restrictions (arrow number 7). However, many federal and state-funded flood control projects, for instance, are designating portions of project budgets specifically to community outreach. Local communities can at times wield significant power and can even block projects in some instances. Local communities have firsthand knowledge and can make valuable observations that impact project design, including information about flood patterns over time, potential erosion areas, habitats needing restoration, and areas of poor infrastructure (arrow number 9). Community outreach also brings a variety of stakeholders to the table and provides space for highly variable and contextual differences.

According to interviewees, although community outreach brings significant benefits to the design process, engaging community stakeholders as participants and collaborators can be challenging. The primary challenges mentioned by participants in interviews are as follows:

First, the design concepts and methodologies used by engineers must be transferred to laypeople in a meaningful way. Translating risk to local communities requires not only strong outreach and speaking skills but also an investment of time and money to teach engineers how to communicate clearly and effectively to the public about risk (arrow number 10). Poor risk communication skills can lead to a lack of interest among the public in communicating with engineers (see Fig 3).

Second, communicating risk to local communities is further challenged when there are language barriers between the engineer(s) and the local community or when economically disadvantaged communities lack access to resources that would otherwise connect them to the project (e.g., internet to access reports, transportation to reach community meetings, etc.). Highlighting the language barrier, participants discussed community education as the most common theme in engagement. They stressed the importance of an informed community and expressed enjoyment in teaching others. While some engineers acknowledged the role of planners in community engagement, views varied. Several participants valued direct involvement in community meetings, noting that relying solely on planners or project managers might not reflect the reality.

**Fig 4:**
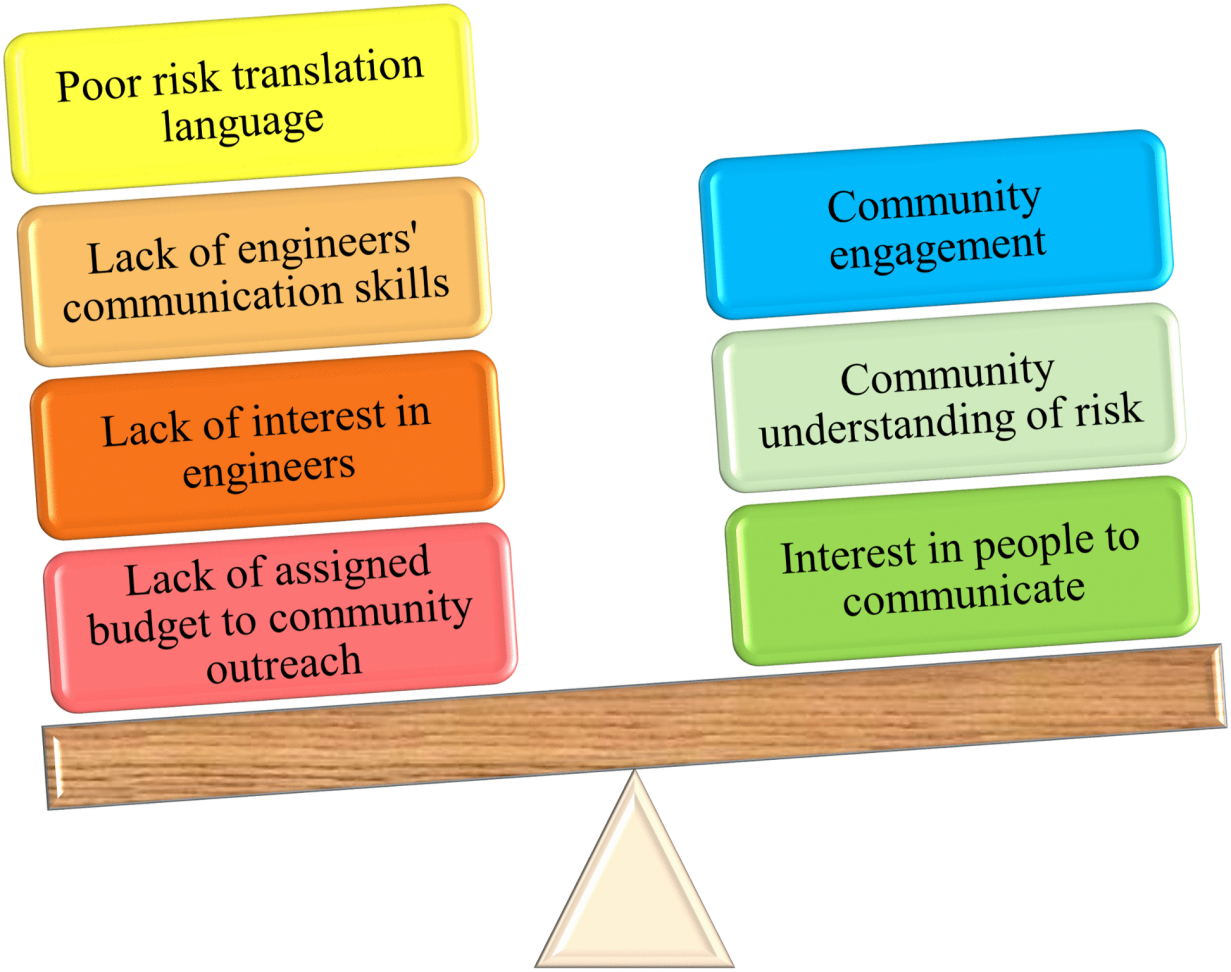
The interaction between community and engineers.

Third, some communities hold strong feelings about their lifestyle, views, easy access to the ocean or other natural resources, etc., and these beliefs may considerably alter outcomes for infrastructure design (arrow number 11). The lack of public knowledge of historical data, probabilistic analysis, and uncertainties surrounding risk make communicating their concerns for decision-making much more difficult. Community members may also not agree with a retreat of infrastructure as a design strategy and consider the retreat a failure. Fig 4 presents key barriers to community interaction from the engineers’ perspective.

To highlight the role of community-level characteristics, many interviewed experts mentioned practical solutions from their point of view, which are outlined below. The suggestions have been grouped according to ease of implementation; thus, those suggestions listed first are deemed the low-hanging fruit. It also should be noted that there is some overlap among the suggestions; however, this is how they were presented by experts, so the overlap is retained.

1. Identifying critical stakeholders for community engagement
2. Including a sociologist in the design process
3. Planning public meetings for community engagement
4. Knowing the audience well
5. Developing social and culturally dependent design frameworks
6. Being aware of the community’s long-term needs
7. Involving the public in projects to understand the risk
8. Prioritizing under-privileged communities
9. Clarifying the benefits of the mitigation scenarios to local communities
10. Improving the interaction among local communities
11. Tackling the equity challenge in community engagement
12. Changing the infrastructure design culture from a standard space to a risk-informed process through community engagement

The findings indicate that while some engineers acknowledge the value of integrating social science into the design process, such collaboration is not yet standard practice in their organizations, which highlights a disconnect between organizational norms and engineers’ personal values. This suggests that while the recognition of this connection is emerging, its implementation remains inconsistent across different organizational contexts. Similarly, while planners in the U.S. are generally acknowledged as key facilitators of interdisciplinary collaboration, participants varied in how they emphasized this role in practice which reflects differences in organizational norms. These diverse perspectives on collaboration appear to be influenced not only by organizational culture but also by factors such as firm size. A number of interviewees also noted the impact of project scope and size on design outcomes. Large projects typically have more funding and resources, which can be used to conduct more precise risk assessments and substantive and meaningful community outreach (arrow number 12). In smaller projects, limited resources may be available for risk analysis and community engagement, which can lead to less accurate risk assessments and less stakeholder involvement. This can result in unanticipated issues arising during the project implementation phase.

According to the findings and supporting quotes presented in the results section, engineers acknowledge certain challenges and shortcomings in the infrastructure design process. The findings highlight that while engineers recognize several challenges in flood infrastructure design—such as regulatory constraints, risk perception, and stakeholder engagement—their perspectives on overcoming these challenges vary. Some participants acknowledged the need for more integrated approaches, including nature-based solutions and enhanced community involvement, yet also expressed concerns about increased complexity, costs, and regulatory hurdles. For instance, while some engineers supported more holistic risk assessments, others noted that stricter requirements or additional stakeholder involvement could slow project timelines and complicate decision-making. This suggests that while awareness of these challenges exists, there is variability in whether engineers see overcoming them as beneficial or as introducing further obstacles to an already complex design process. These findings underscore the tension between innovation and practicality in engineering practice. which reveals that change is not solely a technical issue but also a question of feasibility, resources, and institutional readiness.

### 5.3. Risk tolerance and perception

Axial coding grouped several lower-level open codes together as associated with risk tolerance and perception in the design process. These included financial issues, regulations, personal attitudes (towards risk), environmental factors, and design uncertainties. Interviews revealed significant tensions between the personal risk tolerance of engineers and the norms and conventions of the engineering profession at large. Risk tolerance, while variable from person to person, is heavily driven by organizational culture and regulatory frameworks, shaping design decisions (arrow number 13).

The findings show that the majority of participants tend to be risk-averse, largely influenced by their organizational norms. This caution is further shaped by their strong perception of the potential consequences of taking risks, despite recent advancements in probabilistic risk analysis, as noted by interviewees. Currently, regulatory requirements are shifting from deterministic risk analysis to more probabilistic risk analysis, and this is slowly changing how design decisions are made (arrow number 14). Agencies that adhere to more probabilistic flood risk analysis push engineers to higher levels of risk tolerance. These group- and organizational-level constraints override personal risk tolerance.

The discrepancy among participants suggests that engineers’ views on regulatory effectiveness are shaped not only by their professional roles but also by their exposure to regulatory development processes. Comparatively, engineers with private-sector experience often face the pressure of balancing regulatory compliance with cost constraints. This contributes to a perception that regulations sometimes lag behind best practices. As one noted, private firms frequently operate under business constraints that push them to meet only the minimum regulatory requirements rather than adopt more advanced, risk-informed methodologies—particularly when those methods are more complex or costly. While some federal agencies are indeed moving toward probabilistic design approaches; this shift remains inconsistent across regulatory bodies.

Climate change considerations have begun to lead to new or revised regulations, but further efforts are required in this regard (arrow number 15). The findings suggest that participants view climate change as the most significant source of uncertainty in their design process. This uncertainty is heightened by the perceived lack of adequate integration of climate considerations into existing regulations. As one interviewee noted, effective communication and collaboration between regulatory bodies and engineering professionals are essential to navigate these challenges and ensure that safety standards are both rigorous and feasible to implement within the constraints of real-world engineering projects.

ASCE 7 was recently updated to improve safety standards by moving towards a 500-year design flood standard, which was met with a lot of acceptance within the engineering community. Nonetheless, resistance emerged during drafting mainly due to associated cost concerns. This inner discord highlights the hurdle for new safety regulation implementation (arrow number 16 and 17). Finding ways around financial constraints and regulatory resistance while addressing emerging environmental concerns remains crucial (arrow number 18, and 19).

The findings of this study highlight that while traditional engineering perspectives on infrastructure remain dominant, there is an increasing recognition among practitioners of the importance of nature-based solutions and ecosystem integration in flood infrastructure design. Several engineers acknowledged that environmental factors such as carbon footprints, ecosystem services, and coastal interactions are beginning to be considered in project planning. However, barriers remain, particularly in terms of regulatory adoption, the quantification of nature-based benefits, and economic constraints. Some participants noted that existing frameworks still emphasize deterministic engineering approaches, making it difficult to justify probabilistic, ecosystem-oriented designs within current cost-benefit structures.

These findings suggest that while the perception of infrastructure is evolving, there remains a gap between conceptual advancements in resilience theory and practical engineering applications. Future work should explore how regulatory bodies, engineering education, and interdisciplinary collaborations can further bridge this divide, fostering a more integrated approach to flood infrastructure that balances technical reliability with environmental sustainability.

### 5.4. Personal characteristics of the engineer

Closely linked to an engineer’s risk tolerance are personal characteristics of the engineer, such as attitudes and values. This high-level axial code consisted of lower-level codes of motivations, hindrances, state of the art, and real events.

Importantly for design decisions, some of the interviewees explained that there can be subjectivity in designs, and extreme weather events have taught engineers valuable lessons about the importance of thorough risk assessments before implementing major infrastructure projects. Experiencing extreme flood events may lead individuals to adopt a more conservative approach to design.

The findings show that while organizational norms and external factors influence flood-related solutions, participants’ individual attitudes and motivations also shape their decision-making. Public safety and the multidisciplinary nature of their work were the most common motivations mentioned. Although public safety is a key value for engineers, it can be affected by organizational norms, client demands, and the type of organization. Economic factors, while less frequently mentioned as personal motivations, remain a priority for private sector projects and may limit engineers’ ability to fully apply their own risk perceptions.

The findings show that most participants personally value collaboration with colleagues, beyond organizational norms or interactions with planners, owners, and clients. While they believe they can achieve acceptable designs independently, they see collaboration as key to creating more reliable and sustainable infrastructure that would not be possible otherwise.

Participants noted that the lengthy flood infrastructure design process, coupled with shifts in policies and norms beyond engineers’ control, often leads to demotivation, as these changes can undermine their efforts and influence decisions they cannot influence. The variation in norms across different workplace settings, such as private versus federal sectors, also influences the ethical norms engineers follow. These variations can impact engineering decisions, even though they differ from personal values and beliefs about engineering ethics. It complicates the design process and potentially demotivates engineers when they encounter conflicting expectations.

Additionally, the findings indicate that engineers face challenges in balancing social and environmental ethics, with public safety often prioritized over long-term sustainability. This tension is further complicated by differing perspectives across communities, where some emphasize environmental solutions, while engineers tend to adopt a risk-averse approach, focusing on protecting the area without taking on risks. This highlights the conflict between engineers’ ethical values, such as considering both pro-environmental actions and community needs, and the social or organizational norms within their work environment.

The interviews shed light on the impact of engineers’ personal views and attitudes on their perspectives regarding the perceived gap between current practice and the state-of-the-art. According to the results, personal beliefs significantly influence opinions on the need for changes in state-of-the-art practices. For instance, some engineers may perceive budget and funding constraints, rather than technological limitations, as the primary barriers to advancing infrastructure development. Consequently, this perspective may lead to a diminished emphasis on addressing the gap between current practice and state-of-the-art technologies.

### 5.5. Weak areas of the profession

Finally, interviews revealed a strong desire to strengthen the engineering profession overall and the design of flood infrastructure more specifically by focusing on some of the perceived weak areas of the field. Lower-level codes that comprised this high-level category included training, project maintenance, data, the state of the art, and interorganizational communications. Although this high-level axial code is not a direct influence on the design process, it is an indicator of key areas of concern for many engineers.

The majority of participants identified training and education as the weakest link. Training was highlighted as a broad area in need of improvement and, more specifically, participants noted the need for stronger skills in risk communication. Knowledge transfer is a long-term process. Accordingly, to address issues of risk communication, interviewees believe a sociological grounding should be instilled in the engineering profession. Some participants suggest adding community outreach-related skills to the engineering curriculum. They noted that community engagement lags in flood infrastructure design, while public transit infrastructure has traditionally been stronger at including the community. From a social science perspective, one of the interviewees noted that uncertainties about human behavior in communities – including questions such as – How will people follow evacuation orders? How are our warnings going to be disseminated? - are as consequential as the infrastructure decisions they were working on.

Some interviewees focused on the need for more and better data and a stronger focus on data literacy (among both engineers and non-engineers). For example, one participant explained that *“you must be on your toes with new datasets and work that into the risk analysis more conservatively.”* The importance of data also aligns with the identified gap between state-of-the-art knowledge and practical implementation, which participants recognized as a weakness. They noted inconsistencies between academic progressions and real-world engineering practices, presenting challenges in applying theoretical knowledge and utilizing data effectively for practical solutions.

In addition, the recognition of maintenance as a significant weakness in infrastructure resilience, as highlighted by participants in the interviews, highlights a common concern among industry practitioners. This recognition points to an important area for improvement, indicating a potential gap in current practices. Addressing this weakness requires a reassessment of resource allocation strategies to ensure the long-term sustainability and effectiveness of infrastructure systems. By focusing on maintenance, stakeholders can reduce risks linked to infrastructure deterioration and enhance overall resilience against evolving challenges.

## 6. Conclusion

This exploratory work focused on the following overarching research question: ***What are the emergent sociological and psychological themes driving the design process for flood infrastructure and how do engineers conceptualize, incorporate, and process these themes in their design decisions?***

Using a grounded theory approach four higher-level thematic areas that highlight associations and linkages among key terms and explores the questions, were emerged: 1) Stakeholder involvement and influence (including owners, clients, and communities); 2) Risk perception and tolerance of the engineer (and, specifically, tensions with the tacit norms for risk in the engineer’s home organization and with the profession at large); 3) Personal characteristics of the engineer (such as their attitudes toward disasters based on personal experience), and 4) Key areas of weakness within the profession as a whole.

To conclude on the main themes, some of the key takeaways can be highlighted:

- Effectively integrating community perspectives into engineering decisions is not solely a technical challenge but one deeply shaped by organizational culture, risk perception, and budget constraints
- Engineers in organizations that emphasize cost-effectiveness tend to default to traditional design standards which reinforces a normative preference for budget efficiency over regulatory adaptation or community inputs. In contrast, engineers working in organizations with more flexibility and a culture of innovation reported greater willingness to challenge existing norms and explore alternative solutions, including nature-based infrastructure.
- Public safety is the most widely recognized and prioritized motivation among engineers and reflects a strong personal value. However, this motivation is shaped by client/owner expectations, where profitability plays a more dominant role. These priorities, in turn, influence normative organizational perceptions of risk.
- Engineers’ risk perception is shaped by organizational conservatism and an amplified perception of the consequences associated with risk-taking.
- The most practical approach to successful community engagement is widely recognized as educating the community. Engineers still tend to engage with the community directly, while the role of planners in facilitating this process remains underrecognized.
- Climate change and its inadequate integration into regulatory frameworks are identified as the greatest sources of uncertainty in the design process.
- Engineers primarily define resilience in terms of adaptability, rather than incorporating all its key components into a comprehensive definition. This selective understanding highlights an interesting gap in how resilience is conceptualized in engineering practice.
- Training and education are overwhelmingly regarded as the weakest link in the field. This underscores the need for stronger professional development initiatives.
- Engineers largely focus on the work of undertaking and completing a project; rarely are they afforded the opportunity to reflectively think about the process.

This work has implications for both the research and the engineering communities. Nearly every interviewee commented that they found the interview process engaging and interesting, and that it pushed them to think about their work in different ways. Outcomes from this work can also provide insights as firms and organizations think about organizational culture, strategic planning for the long term, and design infrastructure for a changing climate. Understanding the role of human behavior in the design process is one piece of this complex puzzle. By identifying and understanding engineers’ attitudes, norms, and perceived behavioral controls within their work environments, engineers can take proactive steps towards improving overall design practices. For instance, delving into participants’ insights on the weakest links and their underlying reasons aid to identify similar weaknesses, devise targeted solutions, and contribute to more robust and community-oriented infrastructure development initiatives.

There are some limitations to this study that should be noted. Importantly, the qualitative nature of this work means it is difficult to replicate the study or validate the results. The work presented here is context-specific and repeating such a study among a different group of engineering professionals (e.g., academics), or among those focusing on different infrastructure challenges (e.g., transportation), would likely lead to different results. However, context-specific qualitative work can greatly contribute to gaps in existing research and, when taken in aggregate with other ethnographic and qualitative work can add to knowledge and shape future theory and empiricism. Additionally, the interviews conducted for this study did not explicitly include questions about ethics; this is an important component of the engineering profession, and although our qualitative analysis uncovered a number of ethics-related themes, it would be fruitful to add questions of this nature to future work, and this presents a rich area for future study. It is also important to acknowledge that our study was limited to a specific geographic region of the world and may not fully capture the diversity of experiences and contexts in other settings. Additionally, while our research sheds light on the role of human behavior in the design process, further studies are needed to explore the long-term impacts of these insights on organizational culture and strategic planning. Thus, care should be taken when attempting to generalize these findings to other contexts.

Despite these limitations, this work makes important contributions to research gaps. Such gaps, as noted earlier in the paper, tend to overlook factors that would lead to a more complete understanding of the decision-making process and risk assessment methodologies. By examining these factors, this study uncovers important influences that significantly impact outcomes, highlighting the need for more interdisciplinary projects to consider these aspects. Furthermore, the study identifies a gap in effective community involvement in engineering projects, emphasizing the importance of recognizing factors influencing community engagement.

This study lays the groundwork for building initial qualitative findings and contributes to strengthening interdisciplinary research in this area. However, since the research focuses on the U.S., caution should be exercised when generalizing the results beyond this regional context.

This work also provides a pathway for future research. For instance, with more robust data it may be possible to quantitatively assess the relationships highlighted here. The authors of this work have developed a survey targeted to a larger group of engineers, which is currently underway. Findings from this survey will allow for quantitative analysis, and such results would provide validation for the findings that emerged in this exploratory work. The field would benefit from additional empirical work along these lines, including modeling and statistical analysis that can lend validation to qualitative work.

## Supporting information

S1 File Supplementary material. This file contains multiple supporting appendices (S1-S5). (DOCX)
