# Supplementary material for: Beyond Blueprints: Exploring the influence of social science factors on engineers’ flood infrastructure design decisions
Source: PLoS One. 2026 Mar 27;21(3):e0345154. doi: 10.1371/journal.pone.0345154 (PMC13029751; doi:10.1371/journal.pone.0345154)
Supplement: S1 File — (DOCX) [file pone.0345154.s001.docx]

Supplemental Information

S1 Appendix: interview protocol and methodology

Table S1.1: interview open-ended big picture questions

|  | Interview Protocol |
| --- | --- |
| 1 | First, do you deal primarily with levees or with breakwaters? Tell us a little about your background and training, and your past and current role in dealing with coastal infrastructure. What drove you to this career choice? |
| 2 | What are the strongest motivations/drivers to you personally when you work on (or accept) the design, construction, monitoring, and/or maintenance of coastal infrastructures? How about things that hamper your motivation? |
| 3 | How do you feel about collaborations, teamwork, regular group meetings, and sharing information with other engineers in design, construction, monitoring, and/or maintenance of coastal infrastructure? How much do you value the opinions of your peer engineers? Does their input influence your design decisions and analysis? |
| 4 | How important is it to you to oversee the construction/maintenance and/or track the progress of a project you have designed? How confident are you usually that the projects you design can also be successfully built? What might hinder or hamper this? |
| 5 | How do you feel about local community engagement in design, construction, monitoring, and/or maintenance coastal infrastructures? Do you believe local socioeconomic characteristics have a considerable influence on outcomes for coastal infrastructure projects? |
| 6 | Do you think guidelines, standards, and strategies for coastal infrastructure design, construction, monitoring, and/or maintenance should be changed at all? If yes, how/why? What would be the areas to prioritize or change? (Please elaborate if possible; any examples you have personally experienced?) |
| 7 | What do you think are the major divergences between the state-of-the-art vs practice? How do you think current guidelines or policy get incorporated into the state of the art? |
| 8 | How important is it to consider uncertainties in the design, construction, monitoring, and/or maintenance of coastal infrastructure? In your opinion, what are the most critical uncertainties that engineers need to consider for coastal infrastructures? Why? |
| 9 | What are the key design parameters for (levees OR breakwaters) from your technical point of view? What are the main uncertainties with these parameters? How important are they and how do you actually incorporate these uncertainties in design? |
| 10 | How important do you think it is to consider climate stressors, environmental factors, and coastal ecosystems in coastal infrastructure projects? How about risk? How do you feel about risk in infrastructure design and how do you incorporate it into your designs? |
| 11 | Do you often consider life cycle cost analysis, budget management, insurance plans, and tax rates in design, construction, monitoring, and/or maintenance coastal infrastructures? |
| 12 | What are the most significant resilience components/dimensions for coastal infrastructures—especially levees and breakwaters? Could you walk us through your main goal in designing resilient infrastructure? |
| 13 | If you think about the full cycle of training, design protocols, material and technology, construction, and maintenance, where do you think the weakest link(s) is/are? Which one is behind the others currently? |
| 14 | Are there any other questions or topics you think we should ask other potential interviewees that you think can help us better understand the topic? Do you know any other experts you would recommend we interview? |

S2 Appendix: participants information

Table S2.1: A summary of participant characteristics

| Factor | Number of Participants |
| --- | --- |
| Breakwater | 4 |
| Levees | 4 |
| Dams | 4 |
| Water Resource | 4 |
| Marine Structures | 2 |
| Others (e.g., meteorology, nuclear plants, and Oceanography) | 2 |
| Private Industry | 11 |
| Government | 7 |
| Ph.D. | 8 |
| Masters | 9 |
| Undergraduate | 1 |
| Female | 0 |
| Male | 18 |

Table S2.2: A summary of participant backgrounds

| Factor | Number of Participants |
| --- | --- |
| Coastal\Ocean Engineering | 11 |
| Geotechnical Engineering | 4 |
| Structural Engineering | 4 |
| Water Resources | 3 |
| Environmental Engineering | 1 |
| Aquaculture | 1 |
| Ship Design | 1 |
| Mechanical Engineer | 1 |
| Meteorology | 1 |
|  |  |

S3 Appendix: methodology details

Codes in qualitative data analysis represent interpretation and meaning assigned to data as the initial step of an interview analysis.

It is helpful to briefly elaborate on the process of determining what to code. Broadly, if a component of a transcript is repeated several times, surprises the coder, is explicitly stated as important by the interviewee, has been encountered in previously published research, or aligns with a theory or concept, it is usually deemed relevant to code. Thus, coders act as interpreters, making choices about what to code and what is considered important.

Coding categories were primarily structured around the overarching themes of the interview questions. However, new themes emerged organically based on participants' responses. Some of these themes were identified due to the open-ended nature of the interviews, which allowed participants to elaborate on additional concerns that arose naturally during the discussion.

In grounded theory specifically, coding can typically be categorized into open coding, axial coding, and selective coding.

Coding process includes “open codes” which encompassed Steps 1 and 2. Open coding breaks down large amounts of data (e.g. the full interview transcript) into smaller and more manageable pieces for analysis purposes.

- **Step 1**: In Step 1, we began by reading the transcript and making notes about our first impressions. Then, we re-read the transcript line by line.
- **Step 2**: In Step 2, we began labeling relevant strings such as words, phrases, sentences, or sections in the transcript.

Following open coding, the process continued with more focused and structured approaches. Axial coding builds on open coding by conducting a second analysis of the identified categories in order to reveal the unobserved connections (or axes) among them (Corbin & Strauss, 2008)

- **Step 3**: In Axial coding, step 3, we determined which codes were important and created higher-level categories by combining several codes together. Several themes were created through this process of combining codes. It is important to mention that we did not use all of the codes produced in the previous steps; in fact, many of the initial codes were able to be dropped once categories were combined.
- **Step 4**: In Step 4, we began labeling categories and decided which were the most relevant, ultimately connecting them to each other.
- **Step 5**: To undertake Step 5, we determined if there was a hierarchy among categories or a ranking of importance among the categories in order to identify the primary driver of the work. This core category represented the central thesis of the study.

Through this multi-step process, the coding evolved from initial impressions to a structured representation of key themes and relationships as a foundation for further analysis and interpretation. Interview analysis for grounded theory was conducted using Quirkos qualitative software.

In this study, coders initially created memos and then explored overlaps between memos and pre-defined codes, subsequently refining low-level codes and themes. This approach aligns with grounded theory principles, enabling the construction of conceptual frameworks or theories. Overlap analysis was conducted by first identifying the number of overlaps for each theme and code from the codebook. Themes with significant overlaps were then selected for further investigation. Coders examined the interview segments associated with these paired codes, iteratively exploring overlaps to extract meaningful insights for the results and discussion sections, as well as graphical representations and excluded those which meaningful relations have not been explored.

S4 Appendix: coders’ agreement level quantifications

In this section, the coders’ agreement level quantifications are explained. To calculate coding reliability a coding matrix or table that displays the coding decisions made by each coder for each segment of data is created. This coding matrix used in the calculation is presented in Table X.

- Cohen's kappa coefficient calculation:

Cohen's kappa coefficient is a common measurement to quantify the level of agreement between two coders. To calculate this coefficient, the following parameters are determined:

*Observed agreement* ($P_{0}$): the proportion of segments for which the coders agree on the coding. This is calculated by counting the number of segments where the coders' coding decisions match as follows:

$P_{0}=\frac{Number of agreed segments}{Total number of segments}$ (S4.1)

*Chance agreement* $P_{e}$: the expected agreement between the coders by chance alone. To calculate this, the expected frequency of each code based on the marginal frequencies in the coding matrix is determined. Marginal frequencies are each coder’s decision (assigned code or theme) to a certain segment in interview transcripts. Then, the expected frequency of agreements are computed as follows for each theme:

$E_{{Theme}_{i}}=\frac{Total segments coded as Theme i by Coder 1}{Total segments}\times\frac{Total segments coded as Theme i by Coder 2}{Total segments}$ (S4.2)

${P_{e}=E}_{total}=\sum E_{{Theme}_{i}}$ (S4.3)

*Cohen's kappa coefficient* $\kappa$:

$\kappa=\frac{P_{0}-P_{e}}{1-P_{e}}$ (S4.4)

- Average percentage of agreed segments: this percentage shows in average what is the percentage of the segments that both coders were agreed on.

$P_{agree,ave}=\frac{\sum_{i=1}^{n} \frac{{\# of agreed segments}_{i}}{{Total \# of segments}_{i}}}{n}$ (S4.5)

Table S4.1. Coding matrix and agreement level measurements

| Theme | $n_{seg}^{coded}$ | | $n_{seg}^{agreed}$ | $n_{seg}^{total}$ | $n_{seg}^{not-coded}$ | | $n_{seg}^{both-coded total}$ | $E_{agree}^{total}$ | $P_{agree}^{total}$ | $E_{agree}^{both-coded total}$ | $P_{agree}^{both-coded total}$ |
| --- | --- | --- | --- | --- | --- | --- | --- | --- | --- | --- | --- |
|  | 1 | 2 |  |  | ~2 | ~1 |  |  |  |  |  |
| Risk analysis | 109 | 73 | 49 | 133 | 40 | 7 | 86 | 0.0071 | 0.368 | 0.0149 | 0.57 |
| Community engagement | 108 | 65 | 57 | 116 | 43 | 8 | 65 | 0.0062 | 0.491 | 0.0131 | 0.88 |
| Uncertainties | 79 | 50 | 23 | 106 | 31 | 9 | 66 | 0.0035 | 0.217 | 0.0074 | 0.35 |
| Regulatory | 76 | 72 | 54 | 94 | 15 | 10 | 69 | 0.0049 | 0.574 | 0.0102 | 0.78 |
| Budget and expenses | 63 | 25 | 16 | 72 | 15 | 4 | 53 | 0.0014 | 0.222 | 0.0029 | 0.30 |
| Resilience | 40 | 49 | 26 | 63 | 6 | 9 | 48 | 0.0017 | 0.413 | 0.0037 | 0.54 |
| Collaboration | 51 | 29 | 29 | 51 | 13 | 1 | 37 | 0.0013 | 0.569 | 0.0028 | 0.78 |
| Owner and clients | 45 | 18 | 13 | 50 | 13 | 2 | 35 | 0.0007 | 0.260 | 0.0015 | 0.37 |
| Life cycle cost analysis | 24 | 37 | 12 | 49 | 8 | 5 | 36 | 0.0008 | 0.245 | 0.0017 | 0.33 |
| Background | 25 | 42 | 19 | 48 | 5 | 5 | 38 | 0.0009 | 0.396 | 0.0020 | 0.50 |
| Motivations | 33 | 34 | 21 | 46 | 9 | 0 | 37 | 0.0010 | 0.457 | 0.0021 | 0.57 |
| State of the art vs practice | 35 | 27 | 17 | 45 | 11 | 3 | 31 | 0.0008 | 0.378 | 0.0018 | 0.55 |
| Tracking projects | 43 | 33 | 32 | 44 | 9 | 2 | 33 | 0.0013 | 0.727 | 0.0027 | 0.97 |
| Climate stressors | 32 | 26 | 19 | 39 | 5 | 4 | 30 | 0.0007 | 0.487 | 0.0016 | 0.63 |
| Weakest link | 30 | 26 | 17 | 39 | 11 | 1 | 27 | 0.0007 | 0.436 | 0.0015 | 0.63 |
| Environmental considerations | 32 | 8 | 6 | 34 | 13 | 2 | 19 | 0.0002 | 0.176 | 0.0005 | 0.32 |
| Hamper motivations | 19 | 21 | 16 | 24 | 3 | 0 | 21 | 0.0004 | 0.667 | 0.0007 | 0.76 |
| Total coded segments | | | 1061 |  |  |  | **Notes:** | | | | |
| Total agreed segments | | | 426 |  |  |  | $n_{seg}^{coded}$: # of segments coded | | | | |
| Chance agreement | | | 0.033 |  |  |  | $n_{seg}^{agreed}$: # of agreed segments | | | | |
| Observed agreement | | | 0.40 |  |  |  | $n_{seg}^{total}$: Total # of segments | | | | |
| Cohen's kappa coefficient | | | 0.38 |  |  |  | $n_{seg}^{not-coded}$: # of close by codes or identical | | | | |
| Ave. percentage of agreed segments | | | 0.39 |  |  |  | $n_{seg}^{both-coded total}$: # of segments coded by both coders | | | | |
| Total both-coded segments | | | 731 |  |  |  | $E_{agree}^{total}$ : Expected frequency of agreement | | | | |
| Chance agreement (both-coded) | | | 0.071 |  |  |  | $P_{agree}^{total}$ : Percentage of agreed segments | | | | |
| Observed agreement (both-coded) | | | 0.58 |  |  |  | $E_{agree}^{both-coded total}$: Expected frequency of agreement | | | | |
| Cohen’s kappa coefficient (both-coded) | | | 0.55 |  |  |  | $P_{agree}^{both-coded total}$: Percentage of agreed segments | | | | |
| Ave. percentage of agreed segments (both-coded) | | | 0.58 |  |  |  |  | | | | |

S5 Appendix: detailed results and discussion

**Engineers’ Training Needs vs. the Role of Planners:**

This section provides a detailed account of engineers’ perspectives on interdisciplinary collaboration, including their interactions with planners and involvement in community engagement. Interviews revealed a wide range of attitudes toward interdisciplinary work. Many participants highlighted the importance of involving diverse stakeholders to address complex challenges, emphasizing the critical role of planners. As one participant noted, *“Pretty much everything I do is involved in kind of large stakeholder groups. So absolutely, we need to get all the perspectives at the table to solve some of these challenges related to technical subject matter experts, we need the planners, the environmental scientists, can’t be solved alone.”* Another participant similarly stressed the need for a broad range of expertise, stating, *“And so the understanding of the risk framework is important. And that’s highly multidisciplinary. You need climate experts, you need modelers for coastal engineers. Pretty large spectrum of people that get involved.”*

While engineers generally acknowledged the importance of interdisciplinary collaboration, many indicated that their organizations typically rely on managers to handle external connections. For example, one interviewee mentioned, *“In the core, basically all the community outreach is done through the project manager who’s kinda the face of the project.”* However, this managerial approach does not always ensure effective interdisciplinary engagement. Some engineers described challenges in their work environments, particularly in smaller organizations where dedicated planners are absent, and individuals are expected to assume multiple roles. One participant shared, *“While I worked for a consulting firm, I mentioned it was a small firm and pretty much the engineer was everything. You are the coastal engineer, you are the geotechnical engineer, you are the structural engineer. Honestly, it was a little stressful because I was a meteorologist that kind of switched over into engineering.”*

Several engineers expressed a desire for more direct involvement in interdisciplinary processes and community interactions. This preference reflects a tension between organizational norms—where managers or planners serve as intermediaries—and some engineers’ inclination to engage firsthand. Direct involvement was seen by some as crucial to informed decision-making and aligning project goals with community needs. As one participant explained, *“We need to be present when doing community engagement. Yes, you can do desktop evaluations, but you need to go there because what we see in central planning may not match reality on the ground.”* Another engineer elaborated on the practical challenge of balancing technical objectives with community input: *“As engineers, sometimes we feel like that’s a bother because we’re trying to drive to a goal of designing this robust flow control feature. And we don’t want to be bothered with that. But on the other side of the coin, you really do need to take that into account and know what your client’s goals are and listen to that community feedback.”* Similarly, the complexity of serving both client and public interests was noted: *“Community engagement is designed to serve the community. And sometimes that can flex with what the owner wants to do. We’re serving the owner, and in both cases you might be serving the public—say, serving public safety. So sometimes it can be a little difficult.”*

Despite acknowledging the value of planners, participants rarely mentioned them as active facilitators of interdisciplinary work. Instead, some engineers indicated that community collaboration is inconsistently integrated into their workflows. One engineer remarked, *“We don’t really deal with that,”* while another added, *“The owner usually takes the lead on that.”* These perspectives suggest that organizational structures vary in how they incorporate planners and other interdisciplinary professionals. Engineers also described the inherent difficulties of stakeholder engagement, noting that differing priorities often lead to tension. As one participant stated, *“Every time you involve stakeholders, you’re introducing challenges because everybody pulls to their own side.”* Another observed that learning from failure is sometimes necessary to improve collaboration: *“Unfortunately, you have to get a couple of failures to make people understand.”* Communicating risk and technical solutions to the public posed its own challenges: *“I think what you’re going to see is people reacting differently to their flood protection. On the other hand, people are not receptive to raising the levees because they want to see the ocean.”* Another participant reflected on the difficulty of gaining public acceptance, even when presenting scientific evidence: *“You know, you’re educated people who have science degrees. That’s almost impossible for us to get to where they’ll accept you.”*

Despite these challenges, several engineers expressed enthusiasm for community engagement and found it to be a rewarding aspect of their work. One participant remarked, *“Community engagement is definitely an important part. We have clients from private landowners to state and federal agencies. We work with them directly, trying to help solve their issues.”* Another highlighted the personal satisfaction derived from direct interaction with communities: *“Some of the most fun I’ve had is connecting with the community—thinking you’re making an impact or helping bring a solution to a community problem.”*

Table below presents key points from the interviews that present a range of perspectives on nature-based solutions, ecosystem considerations, and sustainability in infrastructure design.

Table S4.1: Engineers’ Perspectives on Nature-Based and Ecosystem-Friendly Infrastructure

| **Theme** | **Interview Excerpts** | **Key Takeaways** |
| --- | --- | --- |
| **Carbon-Conscious Design** | *"The design life of components, that's obviously very, very important. But also designing systems with respect to carbon is really important too... We really need to take that into consideration and develop new design techniques."* | Some engineers recognize the importance of **carbon footprints** in infrastructure but note that current design techniques may not fully account for it. |
| **Ecosystem Integration in Design** | *"In our state of practice, we do look at the ecosystems in the area. We do look at water, water plants in the area."* | Environmental considerations are already **part of some design processes**, though the extent of their integration varies. |
| **Growing Interest in Nature-Based Solutions** | *"A more meaningful focus on nature-based solutions and integration into the design process would be beneficial."* | Some engineers advocate for **greater integration of nature-based approaches** in flood infrastructure. |
| **Understanding Natural Coastal Systems** | *"The most important part is understanding how coastal environments interact for the greater good… Nature has the ultimate solution, and we need to accept that."* | Some participants emphasize **learning from natural systems** rather than solely relying on engineered solutions. |
| **Living Shorelines as an Alternative** | *"Living shorelines are sometimes classified into another category of projects where it's a little bit more restrictive."* | While some engineers recognize the value of **living shorelines**, existing **regulations may limit their widespread use**. |
| **Challenges in Implementation** | *"We know that nature-based features should work to some extent, but quantifying their cost-benefit impact is a challenge."* | While nature-based solutions are promising, **engineering challenges in quantification and regulatory adoption remain barriers**. |
| **Education and Training Needs** | *"Integrating a holistic approach into the curriculum would definitely be beneficial… Using local resources and coming up with creative designs can help achieve the desired objectives."* | Some engineers see **education and training as key** to advancing **nature-based solutions** in practice. |
